# Stress Affects Central Compensation of Neural Responses to Cochlear Synaptopathy in a cGMP-Dependent Way

**DOI:** 10.3389/fnins.2022.864706

**Published:** 2022-07-29

**Authors:** Daria Savitska, Morgan Hess, Dila Calis, Philine Marchetta, Csaba Harasztosi, Stefan Fink, Philipp Eckert, Peter Ruth, Lukas Rüttiger, Marlies Knipper, Wibke Singer

**Affiliations:** ^1^Department of Otolaryngology, Head and Neck Surgery, Tübingen Hearing Research Centre, Molecular Physiology of Hearing, University of Tübingen, Tübingen, Germany; ^2^Department of Pharmacology, Toxicology and Clinical Pharmacy, Institute of Pharmacy, University of Tübingen, Tübingen, Germany

**Keywords:** long-term potentiation, cochlear synaptopathy, phosphodiesterase 9A inhibitor (PDE9i), cGMP, glucocorticoid, blunted stress response

## Abstract

In light of the increasing evidence supporting a link between hearing loss and dementia, it is critical to gain a better understanding of the nature of this relationship. We have previously observed that following cochlear synaptopathy, the temporal auditory processing (e.g., auditory steady state responses, ASSRs), is sustained when reduced auditory input is centrally compensated. This central compensation process was linked to elevated hippocampal long-term potentiation (LTP). We further observed that, independently of age, central responsiveness to cochlear synaptopathy can differ, resulting in either a low or high capacity to compensate for the reduced auditory input. Lower central compensation resulted in poorer temporal auditory processing, reduced hippocampal LTP, and decreased recruitment of activity-dependent brain-derived neurotrophic factor (BDNF) expression in hippocampal regions (*low compensators*). Higher central compensation capacity resulted in better temporal auditory processing, higher LTP responses, and increased activity-dependent BDNF expression in hippocampal regions. Here, we aimed to identify modifying factors that are potentially responsible for these different central responses. Strikingly, a poorer central compensation capacity was linked to lower corticosterone levels in comparison to those of *high compensators*. *High compensators* responded to repeated placebo injections with elevated blood corticosterone levels, reduced auditory brainstem response (ABR) wave I amplitude, reduced inner hair cell (IHC) ribbon number, diminished temporal processing, reduced LTP responses, and decreased activity-dependent hippocampal BDNF expression. In contrast, the same stress exposure through injection did not elevate blood corticosterone levels in *low compensators*, nor did it reduce IHC ribbons, ABR wave I amplitude, ASSR, LTP, or BDNF expression as seen in *high compensators*. Interestingly, in *high compensators*, the stress-induced responses, such as a decline in ABR wave I amplitude, ASSR, LTP, and BDNF could be restored through the “memory-enhancing” drug phosphodiesterase 9A inhibitor (PDE9i). In contrast, the same treatment did not improve these aspects in *low compensators*. Thus, central compensation of age-dependent cochlear synaptopathy is a glucocorticoid and cyclic guanosine-monophosphate (cGMP)-dependent neuronal mechanism that fails upon a blunted stress response.

## Introduction

Hearing loss has been identified as a common risk factor in cognitive decline and Alzheimer's disease (Lin et al., 2011; Livingston et al., 2017; Montero-Odasso et al., 2020). A direct link has not yet been established (Lin et al., 2011; Fortunato et al., 2016; Uchida et al., 2019; Johnson et al., 2021), although numerous studies on animals and humans point to an interaction of auditory processing and the hippocampus, the part of the limbic system engaged in memory, spatial navigation, and possibly also sensory gating (see for a review: Knipper et al., 2022; Zhang et al., 2022).

A loss of afferent auditory fibers (cochlear synaptopathy) can progress with aging or following “non-traumatic” loud sound, even when audiometric thresholds are normal, and can occur independently of a loss of outer hair cells (OHCs), as shown in aging animals (Kujawa and Liberman, 2009; Sergeyenko et al., 2013; Möhrle et al., 2016) and humans (Bharadwaj et al., 2014; Plack et al., 2014; Viana et al., 2015; Kobel et al., 2017; Liberman and Kujawa, 2017). It has been suggested that deficits in auditory nerve fibers following acoustic trauma and/or age lead to temporal auditory discrimination deficits in animals (Kujawa and Liberman, 2009) and humans (Plack et al., 2014; Liberman and Kujawa, 2017; Wu et al., 2019). Aging people often experience difficulties in perceiving speech in a noisy environment even when audiometric thresholds remain normal (Frisina, 2009; Fullgrabe and Moore, 2014). The development of poor suprathreshold speech processing during aging was attributed to progressive cochlear synaptopathy (Bharadwaj et al., 2014; Bramhall et al., 2015).

We have previously observed that cochlear synaptopathy following acoustic trauma is linked to cognitive changes, including hippocampal long-term potentiation (LTP) or memory acquisition (Matt et al., 2018; Marchetta et al., 2020b). Moreover, temporal auditory processing deficits were not necessarily linked to cochlear synaptopathy when auditory brainstem response (ABR) wave IV amplitudes were disproportionally elevated in response to a reduced ABR wave I. This compensatory mechanism was associated with shorter auditory response latencies and preserved auditory steady state responses (ASSRs), despite age-dependent cochlear synaptopathy (Möhrle et al., 2016). ASSRs are used to investigate the integrity of higher-level auditory structures by measuring the synchronous, phase-locked discharge of auditory neurons to the modulation frequency of acoustic stimuli (Kuwada et al., 2002). The ASSR is a clear indicator for proper temporal processing of amplitude-modulated acoustic stimuli in subcortical areas and in the frontocentral cortex (Engelien et al., 2000). Strikingly, this central compensation response can vary in animals independently of age (Marchetta et al., 2020b); it was observed that, over the range of 9–23 months, mice with high and mice with low central compensation capacity (*high* and *low compensators*, respectively) could be identified (Marchetta et al., 2020b). *Low compensators* had delayed and reduced auditory responses, diminished temporal resolution (shown as reduced ASSR), and decreased hippocampal LTP (Marchetta et al., 2020b). *High compensators* did not show delayed responses to auditory stimuli and had normal hippocampal LTP (Marchetta et al., 2020b). As brain-derived neurotrophic factor (BDNF, *Bdnf* ) highly contributes to hippocampal LTP and is widely expressed in the adult brain, e.g., the hippocampus, amygdala, cerebellum, and cerebral cortex (Hofer et al., 1990; Timmusk et al., 1993), the BDNF-live-exon-visualization (BLEV) reporter mice (Singer et al., 2018b) were used to determine the activity-dependent usage of *Bdnf* exon-IV and -VI promoters through bi-cistronic coexpression of cyan- and yellow-fluorescent-protein (CFP/YFP), respectively. Interestingly, *low compensators* with decreased LTP levels exhibited a lower ability to recruit activity-dependent *Bdnf* exon-IV-CFP and exon-VI-YFP, while *high compensators* with normal LTP exhibited comparatively higher activity-driven *Bdnf* transcript expression (Marchetta et al., 2020b). Searching for a mechanism that distinguishes *high* from *low compensators*, we reconsidered that activity-dependent BDNF expression was also altered in hippocampal capillaries, making differences in hemodynamic responses a possible contributing factor (Marchetta et al., 2020b). This prompted us in the present study to test brain-active vasodilators like the memory-stimulating phosphodiesterase 9A inhibitor (PDE9i) (Kroker et al., 2012). Since the application method itself-a sequence of placebo or drug injections for 10 consecutive days-was a stressful event for the animals, their corticosterone levels were analyzed. We discovered that successful central adaptation to age-related cochlear synaptopathy in *high compensators* is a cGMP- and stress-dependent process that failed in *low compensators* due to their blunted stress response.

## Materials and Methods

### Animals

Animal care, procedure, and experimental protocols correspond to national and institutional guidelines and were reviewed and approved by University of Tübingen, Veterinary Care Unit, and the Animal Care and Ethics Committee of the regional board of the Federal State Government of Baden-Württemberg, Germany. All experiments were performed according to the European Union Directive 2010/63/EU for the protection of animals used for experimental and other scientific purposes. In-house bred mice were kept according to the national guidelines for animal care in a specific pathogen-free animal facility at 25°C on a 12 h/12 h-light/dark cycle, with average noise levels of around 50–60 dB SPL_RMS_.

Female and male homozygous *Bdnf*-live-exon-visualization (BLEV) mice were used and categorized into different age groups. Middle-aged animals were between 9 and 14.7 months, while old animals were between 15.2 and 22.7 months.

The mouse model was generated as described (Singer et al., 2018b). Briefly, the brain-derived neurotrophic factor (BDNF, *Bdnf)* exon-IV and -VI sequences, both including the corresponding promoter sequences, were extended by cyan- and yellow-fluorescent protein (CFP/YFP), respectively, both containing a stop codon. A HA-tag was added to *Bdnf* exon-IV-CFP and a cMyc-tag to *Bdnf* exon-VI-YFP. The translation of the protein-coding *Bdnf* exon-IX was enabled by an internal ribosomal entry site sequence, which keeps the mRNA at the ribosome, despite the presence of a stop codon. Additionally, the growth-associated protein 43 was added to anchor the fluorescent proteins at the site of translation. This allows differential monitoring of the non-coding *Bdnf* exon-IV and *Bdnf* exon-VI by the fluorescent proteins CFP and YFP without interfering with *Bdnf* exon-IX.

### Hearing Measurements

Hearing function was studied before and after treatment with either a placebo or phosphodiesterase 9A inhibitor (PDE9i) by measuring auditory brainstem responses (ABR) and auditory steady state responses (ASSR) in a soundproof chamber (IAC 400-A, Industrial Acoustics Company GmbH, Niederkrüchten), as previously described (Zuccotti et al., 2012; Rüttiger et al., 2013). Hearing measurements were done under anesthesia, 75 mg/kg ketamine hydrochloride (Ketavet^®^, Zoetis GmbH, Berlin, Germany), 5 mg/kg xylazine hydrochloride (Rompun^®^, Bayer Vital GmbH, Leverkusen, Germany), and 0.2 mg/kg atropine (AtropinsulfatB. Braun, Melsungen, Germany), as previously described (Engel et al., 2006; Rüttiger et al., 2013).

The ABR, evoked by short-duration sound stimuli, represents the summed activity of neurons in distinct anatomical structures along the ascending auditory pathway (Burkard and Don, 2007) recorded from subcutaneous electrodes (one active electrode at the recorded ear, the reference electrode at the vertex, and the grounding electrode at the caudal third of the back of the animal, Supplementary Figure 1A). A microphone (Bruel & Kjaer 4939, Naerum, Denmark) was used to calibrate and record the acoustic stimuli. ABR thresholds were elicited with a phase-alternating click (100 microsecond duration with an FFT mean of 5.4 kHz), noise-burst (1 ms duration, FFT mean of 7.9 kHz), or pure-tone stimuli (3 ms duration, including a 1 ms cosine squared rise-and-fall envelope, 2–32 kHz). The stimulus level was increased stepwise from 10 to 100 dB SPL in 5dB steps. The stimuli were generated with an I-O-card (PCIe-6259, National Instruments, Austin, Texas, USA) in an IBM compatible computer. The SPL of the stimuli was modulated by custom-made amplifier and attenuator systems (Wulf Elektronik, Frankfurt). The measured signals were bandpass filtered from 200 Hz to 5 kHz (F1, 6-pole Butterworth hardware filter, Wulf Elektronik) and amplified by 100,000. The analog/digital (A/D) rate was 20 kHz. Each stimulus had a recording interval of 16 ms and was directly repeated and averaged up to 512 times (256 for pure-tone stimuli).

ASSRs were measured with amplitude-modulated sinusoidal stimuli (Supplementary Figure 1B), with a carrier frequency of 11.3 kHz, a modulation frequency of 512 Hz, and a maximal (100%) modulation depth. For the growth function, the amplitude-modulated stimuli were presented between –10 and 60 dB relative to the threshold in 5 dB steps. The response on amplitude modulation was tested on the ear with the lower click- and noise-evoked threshold directly after finishing the standard ABR protocol using the same electrode positions (Supplementary Figure 1A). ASSR responses to amplitude-modulated tones were recorded in epochs of 1,114 ms, filtered (50–2,000 Hz 6th order band-pass Butterworth), amplified (80 or 100 dB), and sampled at 50 kHz by 16-bit A/D conversion of a 5 or 10 V input range by the I-O-card (National Instruments).

### Phosphodiesterase 9A Inhibitor

3 mg of PDE9i (BAY 73-6691, Bayer Vital GmbH, Leverkusen, Germany) was dissolved in 500 μl EtOH and diluted with 9.5 ml of 10% Solutol (BASF, Mannheim, Germany). The placebo solution consisted of a dilution medium without PDE9i.

The placebo or PDE9i was administered to the mice intraperitoneally for 10 consecutive days at approximately the same time.

### Field Excitatory Postsynaptic Potential Recordings in Hippocampal Slices

Extracellular field excitatory postsynaptic potential (fEPSP) recordings were performed according to standard methods as previously described (Matt et al., 2011; Chenaux et al., 2016).

In brief, 400 μm-thick coronal brain slices were cut on a vibratome (Leica VT 1000S) in an ice-cold dissection buffer (mM): 127 NaCl, 1.9 KCl, 1.2 KH_2_PO_4_, 26 NaHCO_3_, 10 D-glucose, 2 MgSO_4_, and 1.1 CaCl_2_, constantly saturated with 5% CO_2_ and 95% O_2_ (pH 7.4). Slices were incubated in oxygenated artificial cerebrospinal fluid (in mM: 127 NaCl, 1.9 KCl, 1.2 KH_2_PO_4_, 26 NaHCO_3_, 10 D-glucose, 1 MgSO_4_, 2.2 CaCl_2_; pH 7.4) for 1 h at 30°C and, afterwards, stored at room temperature. Recordings were performed in a submerged-type recording chamber (Warner Instruments). Stimulation (TM53CCINS, WPI) and recording (artificial cerebrospinal fluid-filled glass pipettes, 2–3 MΩ) electrodes were positioned in the stratum radiatum to record Schaffer collateral fEPSPs. Signals were amplified with an Axopatch 200B (Molecular Devices), digitized at 5 kHz with an ITC-16 (HEKA) and recorded using WinWCP from the Strathclyde Electrophysiology Suite. The stimuli (100 μs) were delivered through a stimulus isolator (WPI). For each individual slice, the strength of the stimulation (typically between 30 and 125 μA) was chosen to evoke 40–60% of the maximal response, defined by an initial fEPSP slope. Only slices that showed stable fiber volley (FV) and fEPSP were used for further recording. The same stimulus intensity was applied during baseline recording (0.067 Hz, 20–30 min) and induction of long-term potentiation (LTP) using 100 stimuli during 1 s (100 Hz, 1 s).

### Tissue Preparation

Tissue preparation was carried out as described in detail previously (Singer et al., 2016; Matt et al., 2018). In brief, brains were dissected and fixed in 2% paraformaldehyde for 48 h. For cochlear cross-section immunohistochemistry, cochleae were isolated, fixed by immersion in 2% paraformaldehyde, 125 mM sucrose in 100 mM phosphate-buffered saline (pH 7.4) for 2 h and then decalcified for 45 min in RDO rapid decalcifier (Apex Engineering Products Corporation, Aurora, IL, USA). Cochleae were stored in Sucrose-Hank's solution rotated at 4°C overnight before they were embedded in Tissue-tek and cryosectioned in slices of 10 μm, and mounted on SuperFrost^*^/plus microscope slides before storage at –20°C.

### Immunohistochemistry

Immunohistochemistry was carried out as described in detail previously (Singer et al., 2016). Antibodies against C-terminal-binding protein 2 (CtBP2)/RIBEYE (rabbit, diluted 1:750; ARP American Research Products, Inc.™, Waltham, MA, USA) to detect ribbons (Khimich et al., 2005), neurofilament 200 (NF-200, mouse, 1:3,000; Sigma-Adlrich, St. Louis, MO, USA), parvalbumin (PV, rabbit, diluted 1:2,000; Abcam, Cambridge, UK), Synaptobrevin 2 (mouse, diluted 1:200; Synaptic Systems, GmbH, Berlin, Germany), and desmin (mouse, diluted 1:100; Abcam, Cambridge, UK) were used. Primary antibodies were detected using appropriate Cy3 (1:1,500, Jackson Immuno Research Laboratories, West Grove, PA, USA) or Alexa488 (1:500, Invitrogen Molecular Probes, Paisley, UK) secondary antibodies.

All samples were viewed as previously described (Zampini et al., 2010) using an Olympus BX61 microscope (Olympus, Hamburg, Germany), equipped with epifluorescence illumination and analyzed with CellSens Dimension software (OSIS GmbH, Münster, Germany). To increase spatial resolution, the slices were imaged over a distance of 15 μm within an image stack along the *z*-axis (z-stack), followed by 3-dimensional deconvolution using CellSens Dimension's built-in algorithm.

### Corticosterone Analysis

Corticosterone concentration in the blood was assessed using the corticosterone ELISA kit (Catalog Nr. ADI-901-097) from Enzo Life Science Inc. (Farmingdale, NY, USA). An assay buffer and a wash buffer were prepared. The assay buffer solution is prepared by diluting the assay buffer provided (a tris buffered saline containing proteins and sodium azide as a preservative) 1:10 with deionized water; subsequently, a wash buffer solution is made by diluting the wash buffer provided (a tris buffered saline containing detergent) 1:20 with deionized water. Afterwards, 5 standard solutions are prepared: 200,000 pg/ml corticosterone standard solution is diluted 1:10 with the assay buffer in a plastic tube, and serial dilutions are made to have these final concentrations of corticosterone 20,000, 4,000, 800, 160, 32 pg/ml, respectively. Each tube is mixed completely using a vortex mixer (MS2 Minishaker, IKA™, Wilmington, NC, USA) to have a correct concentration. Then, the wash buffer solution is made by diluting the wash buffer provided 1:20 with deionized water. Later, wells (covered with a donkey antibody specific to sheep IgG) are disposed on the microtiter plate (Costar™). 100 μl of standards is pipetted into the appropriate wells. 100 μl of samples previously centrifuged 3,000 x g for 5 min with Heraeus Pico 17 Microcentrifuge (ThermoFisher Scientific, MA, USA) and diluted 1:20 with the assay buffer is pipetted into the specific wells and is run in duplicate. Therefore, 150 μl of the assay buffer solution is pipetted into non-specific-binding (NSB) wells, and 100 μl of the assay buffer is pipetted into positive-control (B_0_) wells. NSB and B_0_ wells will give a 0% and a 100% binding, respectively, with the antibody when the enzyme conjugated with corticosterone is added into the wells.

Then, 50 μl of corticosterone ELISA Conjugate (a solution of alkaline phosphatase conjugated with corticosterone), which competes with corticosterone in samples and standards to the antibody bound, is pipetted into each well, except the total activity (TA) and blank wells. 50 μl of corticosterone ELISA Antibody (a solution of sheep polyclonal antibody) is pipetted into each well, with the exception of the blank, TA, and NSB wells. Next, the plate is incubated at room temperature and shaken at 300 rpm for 2 h using a Rotamax 120 shaker (Heidolph Instruments, Schwabach, Germany). After that, the wells are washed three times with 400 μl of wash buffer solution per well. 5 μl of corticosterone ELISA Conjugate is pipetted into the TA wells, and 200 μl of the p-nitrophenyl phosphate solution is pipetted into every well. Afterwards, the plate is incubated at room temperature for 1 h without shaking. 50 μl of Stop Solution (trisodium phosphate in water) is added to each well, and the plate is read immediately at 405 nm. The four-parameter logistic curve works as the following: before making the standard curve, the average of the optic density of each duplicate is subtracted from the optic density average of the NSB to obtain the optic density net values, which are then divided by the net B_0_ to obtain the percentage binding.

### Experimental Design

Hearing function was analyzed 3–5 days prior to placebo or PDE9i application and 1–3 day(s) after the last injection. The animals were separated into one group analyzed for LTP and another group for molecular analyses of brain slices. Blood was taken in the first 15 min of the first anesthesia before and after the 10-day injection protocol. The animals were prepared 1 day after the last hearing measurement. Details for *n* numbers of the single experiments are given in Supplementary Table 1.

### Data Analyses

#### Statistics and Numbers

Unless otherwise stated, all data were presented as group mean with standard error of the mean (SEM) for *n* animals per the experimental group. Data were tested for normal distribution (the Shapiro-Wilk normality test, α = 0.05). Differences of the means were compared for statistical significance either by ungrouped two-tailed Student's t-test (parametric)/ Mann–Whitney U test (non-parametric), 1-way, or 2-way ANOVA (parametric)/ Kruskal–Wallis test (non-parametric) with α = 0.05 and correction for type 1 error after Sidak's test/the Bonferroni's multiple comparisons test (parametric) or the Dunn's multiple comparison's test/the two-stage linear step-up procedure of Benjamini, Krieger, and Yekutieli (non-parametric). In figures, significance is indicated by asterisks [trend (^*^) *p* ≤ 0.1, ^*^p < 0.05, ^**^p < 0.01, ^***^p < 0.001, ^****^p < 0.0001]. ns denotes non-significant results (*p* > 0.1). All statistical information and *n* numbers can be found in the figure legends and in Supplementary Table 1. Statistical calculations and visualizations were done using GraphPad Prism.

#### ABR Analysis

The ABR evoked by the click, noise-burst, and pure-tone frequency-specific auditory stimuli was examined as described (Marchetta et al., 2020a). The threshold for all click, noise, and pure-tone ABR measurements was manually defined as the lowest sound level at which a clear signal could be discriminated from the baseline (Valero et al., 2017).

**Figure 1:**
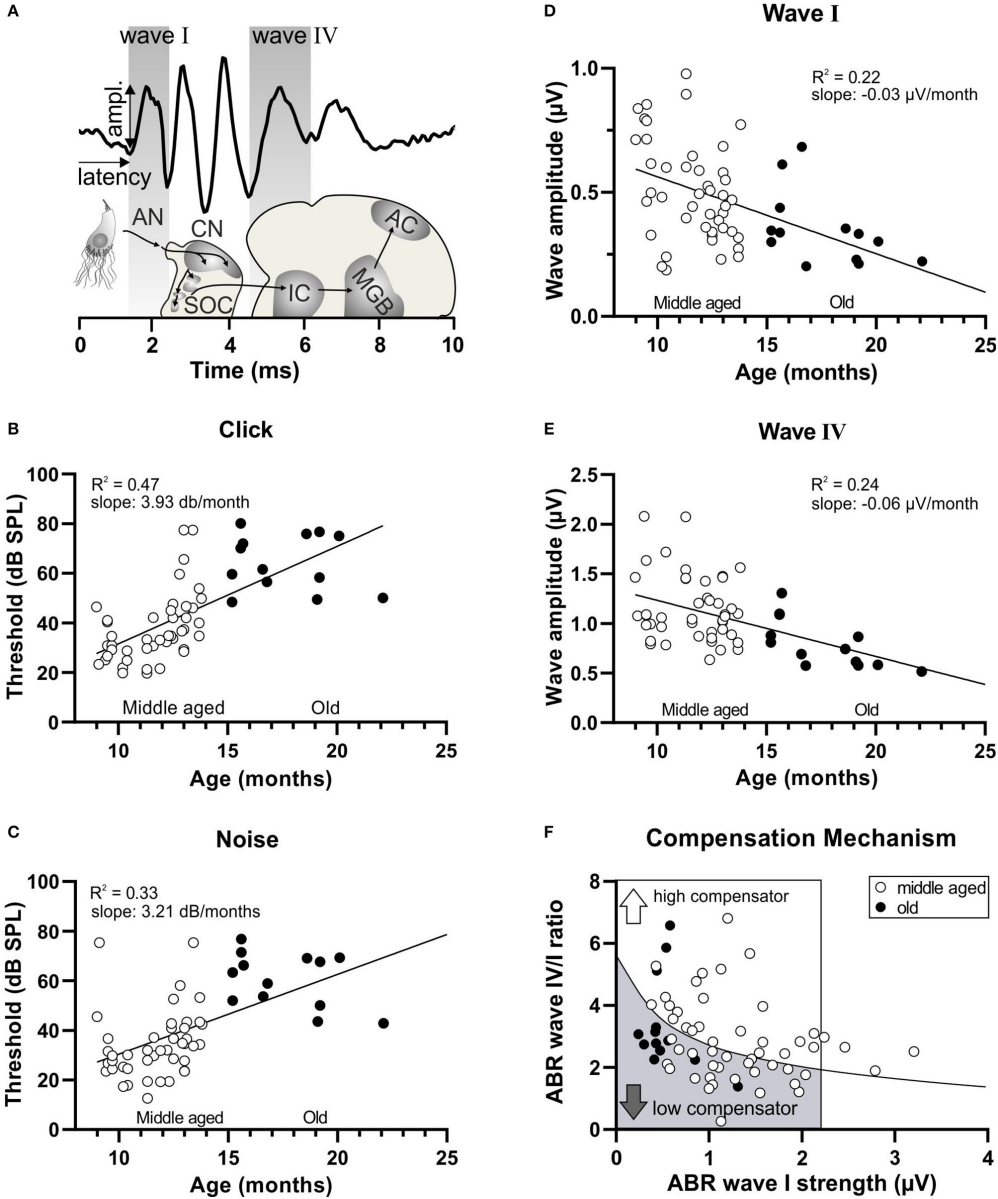
Decrease in hearing function over age and subsequent subdivision by the compensation mechanism. **(A)** Schematic drawing of the auditory pathway and stimulus-evoked deflections of ABR waves correlated with anatomical structures. AN, auditory nerve; CN, cochlear nucleus; SOC, superior olivary complex; IC, inferior colliculus; MGB, medial geniculate body; AC, auditory cortex. **(B)** In a homogenous group of aging animals (white circles < 15 months, middle aged; black circles > 15 months old), increasing age was significantly correlated with an increasing threshold in response to click stimuli and **(C)** noise stimuli. **(D)** In addition, increasing age was significantly correlated with a decreasing amplitude of ABR wave I **(E)** and ABR wave IV. **(F)** A schematic diagram representing animals with reduced ABR wave I (inside the box; ABR wave I < 2.19 μV) subdivided by the black regression line, depending on their central compensation capacity into low compensators (the lower wave IV/I ratio) and high compensators (the higher wave IV/I ratio).

ABR waveforms (Figure 1A) were analyzed for consecutive amplitude deflections (peaks), with each wave consisting of a starting negative (n) peak and the following positive (p) peak. Peak amplitudes of ABR waves I and IV were extracted in the present study and defined as follows: wave I: In-to-Ip (0.8–1.8 ms), the summed activity originating from the auditory nerve (Johnson and Kiang, 1976); and wave IV: IVn-to-IVp (4.1–4.9 ms), the synchronous neural activity that arises from the lateral lemniscus and inferior colliculus (IC) (Melcher and Kiang, 1996). A customized program (“PEAK.exe”) was used to extract ABR peaks on the basis of these definitions. ABR peak-to-peak (wave) amplitude growth functions were constructed for individual ears on the basis of the extracted peaks. ABR wave amplitude growth functions were calculated for increasing stimulus levels with reference to the ABR thresholds (from 0 to a maximum of 80 dB above the threshold in 5 dB steps). Statistical analysis was performed in a range of 20–55 dB relative to the threshold to ensure a consistent number of data points for all treatment conditions and time points.

In Figures 1D,E, the peak input-output function for amplitude of the click-ABR measurements was averaged for intensities between 0 and 40 dB relative to threshold (re thr) for each individual ear and analyzed as previously described (Chumak et al., 2016).

For further analysis, the *strength* of the click-ABR growth function was determined in 5 dB steps until a maximum of ca. 105 dB SPL. Therefore, the three highest amplitude values of the individual ears' supra-threshold amplitude growth function were averaged [described in more detail in Singer et al. (2018a)].

The wave IV/I ratio was calculated using click-ABR data for individual animals at all intensities re thr; the ABR wave IV amplitude was divided by ABR wave I amplitude. For further analyses in Figure 1F, the mean between 0 and 40 dB (re thr) was calculated.

To determine the level of central compensation for individual animals, the wave IV/I ratio was plotted against wave I strength (Figure 1F), as previously described (Marchetta et al., 2020b). The power function (y = a^*^x^*b*^) was inserted as a regression line for each group. Only the animals with wave I strength smaller than 2.19 μV [which is the general wave I strength of all the mice corrected by the limitation value of previous studies (Marchetta et al., 2020b)] were subdivided along the black regression line (each mouse is represented by the average of two ears). The animals below the line were defined as *low compensators*, while the animals above were defined as *high compensators*. The animals that were lying on the line were removed from further analysis.

#### ASSR Analysis

Response trials (Supplementary Figure 1C) from 32 artifact-cleaned epochs of every condition were averaged and spectral power determined by Fast Fourier Transformation (FFT; Supplementary Figure 1D). From the spectrum of either condition, the signal response was determined as the spectral peak within a frequency window of ± 8 Hz (range, 16 Hz) around the modulation frequency (first harmonic). Noise levels were determined within the same frequency window using an iterative procedure. For this, first, we calculated the average and standard deviation of the spectral power from the lowest 2 and the highest 2 bins of the 16 Hz-wide FFT window around the modulation frequency. Then, we calculated z scores for each next-highest and next-lowest bin within the FFT window. If the spectral amplitude of both bins fell within the confidence ranges around the noise average (criterion = 1.96), the two bins were enclosed to the new mean and standard deviation. If the z-score of one of the newly considered bins exceeded the criterion, the iterative calculation was aborted and the resulting average and variance of the noise reported for subsequent analyses. Dependent on the spectral noise, on average, 14 points (range, 4–42) did contribute to the calculation of the noise level.

Epochs from alternating and non-alternating-phase stimulus presentation contributed to the average. No differences of spectral amplitudes were found for the alternating-phase stimuli, indicating no residual cochlear microphonic or temporal fine structure responses were confounding the spectral data at the first harmonic of the response. We analyzed results for the first to 6th harmonic (Supplementary Figure 1D). We show results for the first harmonic only. Summation of all 6 harmonic amplitudes yielded a result similar to the amplitude for the first harmonic.

#### fEPSP Recordings in Hippocampal Slices

The fEPSP baseline was determined by averaging fEPSP initial slopes from the period before the tetanic stimulation (at least 15 min of stable recording). The level of LTP was determined by averaging fEPSP slopes from the period between 50 and 60 min after the high-frequency stimulation (HFS). Before the tetanic stimulation, each slice was used to record input-output relationship (IOR, 25–150 μA in 25 μA steps) and paired-pulse facilitation (PPF, 10–20–50–100–200–500 ms interstimulus interval (ISI) at the same stimulation strength as LTP recordings). IOR changes in the fEPSP slope and FV amplitude were normalized within each slice (% from the maximal response at the highest stimulus strength was calculated), and averaged values for each group were plotted against the stimulus intensity. The PPF paired-pulse ratio of the fEPSP2/fEPSP1 slope and amplitude at each ISI was defined per slice, and mean values per group were plotted. fEPSP1 was calculated as an average of fEPSP1s from all interstimulus intervals for each single slice. Four traces were averaged for each single data point analyzed. Data were analyzed and processed using Clampfit 10 (Molecular Devices) and Microsoft Excel.

The data presented per experimental group/condition contained (in addition to mean ± SEM) single dots, showing the fEPSP slope values for each individual brain slice. The *n* indicates the number of slices and animals (slices/animals) used in the analysis (**Figure 6**).

#### Fluorescence Analysis of Immunohistochemistry

For inner hair cell (IHC) ribbon counting, pictures were taken from all turns of both ears from duplicate immunohistochemical staining.

Pictures acquired from brain sections stained for PV were analyzed using the free Image J software (NIH, Bethesda, MD, USA). For each section, pictures for each single channel (YFP, CFP, PV) were saved and analyzed independently. For the 10× magnified pictures, after conversion to an 8-bit image, background was reduced using the rolling ball algorithm (available as a tool for Image J), with standard parameters in each single channel picture. Afterwards, the integrated density of the fluorescence of CFP, YFP, and PV within the picture was calculated. For each individual animal, both hippocampus hemispheres were analyzed by taking a picture from each side.

#### Corticosterone Analysis

Calculation of the corticosterone levels was performed using an online data analysis tool (myassays.com). Finally, corticosterone levels were averaged per group/treatment condition and presented as mean ± SEM.

## Results

### Animals With Age-Related Hearing Loss Differ in the Ability to Centrally Compensate Cochlear Synaptopathy

Previous and present findings document that aging mice could be subdivided into two groups with a higher or lower ability to centrally compensate for a diminished auditory nerve response through a larger or smaller suprathreshold ABR wave IV/I ratio, respectively, and through elevated or attenuated responses to amplitude-modulated tones (Marchetta et al., 2020b). An additional group of 58 animals, subdivided into middle-aged (9–14.7 months, Figure 1, white circles) and old (15.2–22.7 months, Figure 1, black circles) mice, was analyzed using lower frequency, containing (click) and higher frequency containing (noise-burst) stimuli. Hearing thresholds (Figures 1B,C) and the fine structure of suprathreshold ABR waves corresponding to activity in the auditory nerve (wave I, Figures 1A,D) and lateral lemniscus and IC (wave IV, Figures 1A,E) were analyzed. Upon plotting thresholds to click or noise-burst stimuli as a function of age, we observed a linear correlation (Figure 1B, linear regression, Y = 3.392^*^X−7.781, *R*^2^ = 0.047, *p* < 0.0001, *n* = 58 mice; Figure 1C, linear regression, Y = 3.212^*^X−1.677, *R*^2^ = 0.33, *p* < 0.0001, *n* = 58 mice), indicating that thresholds increased with increasing age. When plotting ABR wave I or wave IV amplitude as a function of age, we observed a significant negative linear correlation, indicating that auditory nerve and brainstem activity decreased with increasing age (Figure 1D, linear regression, Y = –0.03099^*^X + 0.08725, *R*^2^ = 0.22, *p* = 0.0002, *n* = 58 mice; Figure 1E, linear regression, Y = −0.05651^*^X + 1.798, *R*^2^ = 0.24, *p* < 0.0001, *n* = 58 mice). To define the individual level of compensation, we plotted the ABR wave I strength (average of the three highest wave amplitude values) against the ABR wave IV/I ratio (Figure 1F) (see methods). The power function (y = a^*^x^*b*^) inserted as a regression line was calculated using all available measurements from this mouse line in order to ensure a robust database. The animals characterized by dots lying below the black regression line were assigned to the category *low compensators* (Figure 1F, gray arrow), while the animals characterized by dots lying above the black regression line were assigned to *high compensators* (Figure 1F, white arrow). We confirmed previous findings (Marchetta et al., 2020b) that both *high* and *low compensator* groups included middle-aged and old mice (Figure 1F, bottom, white and black circles). Due to the limited *n*-number in the group of the old mice, especially for the *high compensators* (*n* = 3), we decided to proceed only with the group of the middle-aged mice of *n* = 18 *low compensators* and *n* = 26 *high compensators*.

In conclusion, the process of aging brings about a worsening of hearing function, here reflected in increasing thresholds and decreasing ABR wave amplitudes; however, central compensation abilities are independent of age.

### *Low Compensators* Exhibit Reduced ASSR Response to Amplitude-Modulated Tones

**Figure 2:**
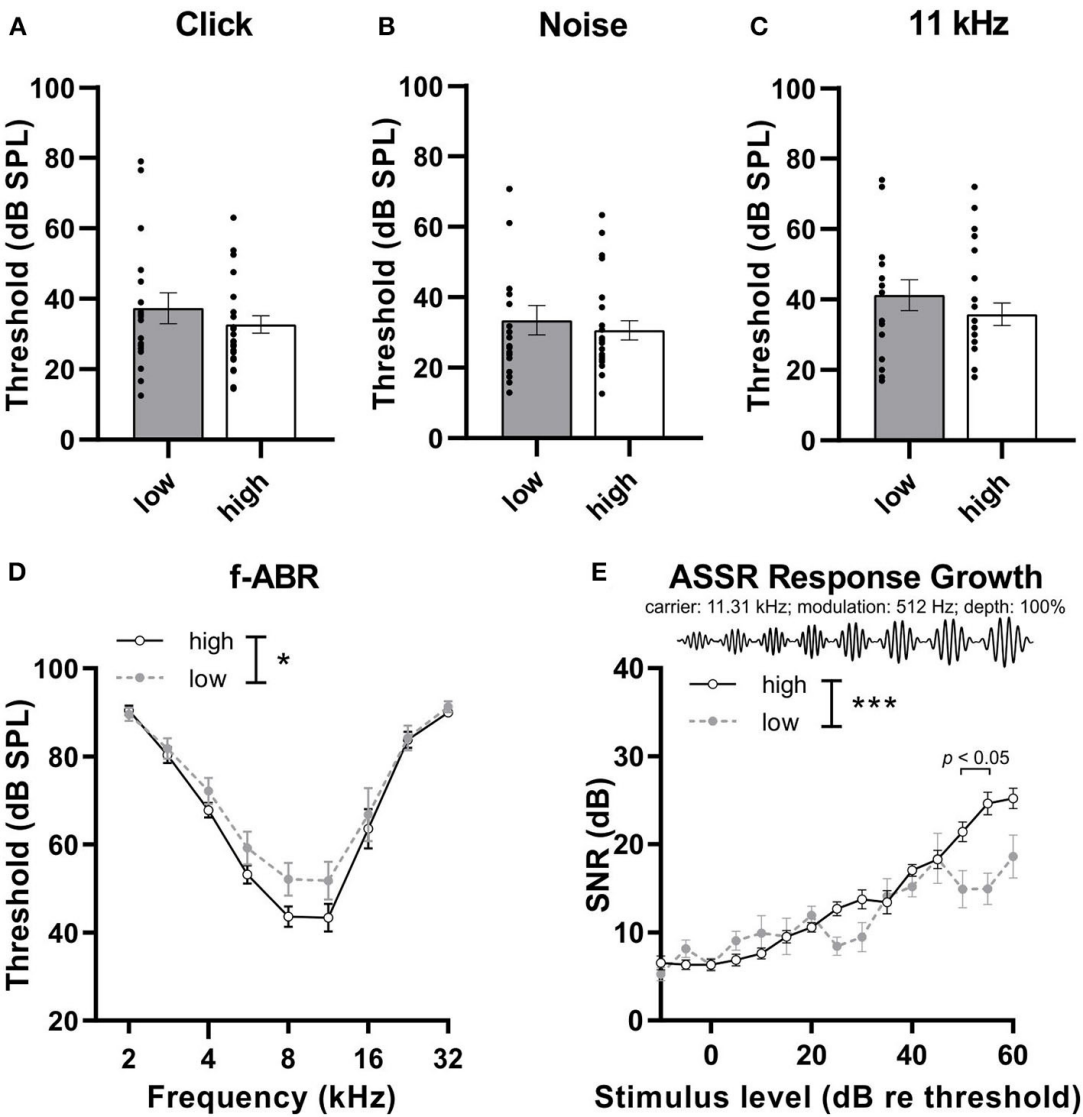
The hearing phenotype of low and high compensators before treatment. **(A)** Low and high compensators showed similar thresholds in response to click and **(B)** noise stimuli. **(C)** High and low compensators showed similar thresholds in response to 11 kHz pure-tone stimuli. **(D)** However, with pure-tone, frequency-specific auditory stimuli (2-32 kHz), low compensators showed elevated thresholds compared to high compensators. **(E)** The temporal auditory resolution was significantly decreased in low compensators in comparison to high compensators. Re threshold, relative to threshold. Mean ± SEM. *p <0.05, ***p <0.001, 2-way ANOVA.

To determine whether the distinction between *high* and *low compensators* is independent of OHC function, hearing thresholds were analyzed and compared. No differences were observed in the thresholds of *high* and *low compensators* in response to click [Figure 2A, Mann–Whitney U test, U (30.05, 34.55) = 205.5, *p* = 0.50, low: *n* = 18, high: *n* = 26 mice], noise-burst [Figure 2B, Mann–Whitney U test, U (27, 27.35) = 200.5, *p* = 0.70, low: *n* = 18, high: *n* = 26 mice], or 11 kHz pure-tone stimuli [Figure 2C, Mann–Whitney U test, U (33, 43) = 197.5, *p* = 0.39, low: *n* = 18, high: *n* = 26 mice]. However, in response to the pure-tone stimuli of increasing frequencies (2–32 kHz, half-octave steps), we observed an increased threshold in *low compensators* in comparison to *high compensators* [Figure 2D, 2-way ANOVA, *F*(1, 325) = 5.00, *p* = 0.03, Bonferroni's multiple comparisons test, low: *n* = 18, high: *n* = 26 mice]. The hearing phenotypes of *high* and *low compensators* were further characterized by analyzing the auditory temporal resolution through using amplitude-modulated stimuli to obtain ASSRs. In the present study, the growth of the ASSR to amplitude-modulated tones with increasing loudness was tested (carrier frequency, 11.32 kHz; modulation frequency, 512 Hz; modulation depth, 100%) (Figure 2E). In the ASSR response-growth function, *low compensators* had a significantly reduced signal-to-noise ratio (SNR) in comparison to *high compensators*, particularly at high sound pressure levels levels [Figure 2E, 2-way ANOVA, *F*(1, 546) = 13.41, *p* = 0.0003, Bonferroni's multiple comparisons test, low: *n* = 14, high: *n* = 20 mice].

In conclusion, *high* and *low compensators* do not differ in the threshold, except to pure-tone stimuli, where *low compensators* seem to have higher thresholds in comparison to *high compensators*. In addition, *low compensators* show a temporal auditory processing deficit, particularly for high sound pressure levels.

### *Low Compensators* Exhibit a Reduced Stress Response to Injection-Induced Stress That Is Independent of Hearing Thresholds

**Figure 3:**
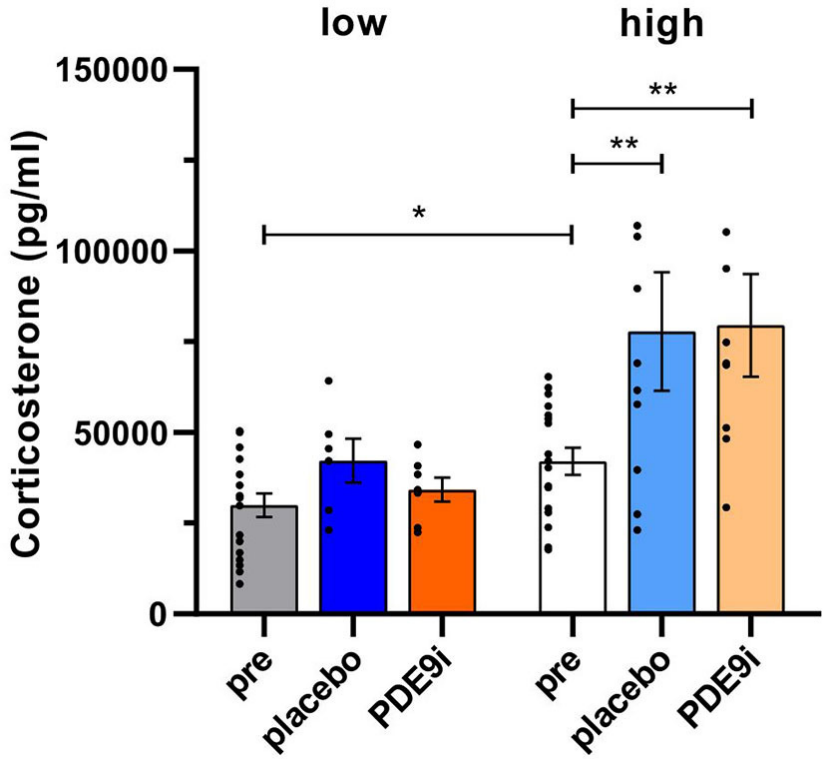
Corticosterone levels in low and high compensators before and after treatment with either placebo or PDE9i. Prior to treatment, low compensators had lower corticosterone levels in comparison to high compensators. Significantly elevated corticosterone levels were found only in high compensators but not in low compensators after treatment with either placebo or PDE9i. However, no significant differences were observed between the two post-treatment conditions in either high or low compensators. Mean ± SEM. *p < 0.05 (two-tailed t-test pre low vs. pre high), **p < 0.01 (the Bonferroni's multiple comparison test for high compensators).

We next compared the effect of PDE9i in comparison to a placebo injection on 10 subsequent days. To test whether the placebo treatment alone exerted a possible stress effect that influenced central neuronal activity, we measured the blood-corticosterone levels before and after treatment. Interestingly, *low compensators* showed significantly lower pre-treatment corticosterone levels in comparison to *high compensators* [Figure 3, gray bar vs. white bar, two-tailed Student's t-test: *t* (35) = 2.42, *p* = 0.02, low pre: *n* = 18, high pre: *n* = 19 mice]. Following 10 days of injection with either placebo or PDE9i, a significant increase in blood-corticosterone levels was observed in *high compensators* after both placebo (Figure 3, white bar vs. light blue bar), and PDE9i injection [Figure 3, white bar vs. light-orange bar, 1-way ANOVA, *F*(5, 63) = 6.79, *p* < 0.001, Bonferroni's multiple comparisons test, low pre: *n* = 18, high pre: *n* = 19, low placebo: *n* = 6, low PDE9i: *n* = 7, high placebo: *n* = 10, high PDE9i: *n* = 9 mice]. *Low compensators* did not show significant changes in blood-corticosterone levels, following 10 days of injection with either treatment (Figure 3, gray bar vs. dark blue bar or dark-orange bar).

As no significant differences between PDE9i or placebo treatment were observed in either *low* or *high compensating* animals, it can be inferred that it is not PDE9i that influences blood corticosterone levels but, rather, the injection itself.

To determine whether the altered stress response between *high* and *low compensators* was reflected in differences in hearing thresholds after treatment, click and noise-burst ABRs were measured (Supplementary Figure 2). Neither the injection itself nor treatment with PDE9i had an effect on ABR thresholds for click [Supplementary Figure 2A, 1-way ANOVA, *F*(3, 24) = 0.19, *p* = 0.91, pre (gray), and post placebo (dark blue): *n* = 6, pre (gray) and post PDE9i (dark orange): *n* = 8 mice; Supplementary Figure 2B, 1-way ANOVA, *F*(3, 36) = 0.63, *p* = 0.60, pre (white) and post placebo (light blue): *n* = 9, pre (white) and post PDE9i (light orange): *n* = 11 mice] and noise-burst stimuli [Supplementary Figure 2C, Kruskal–Wallis test, H (3) = 0.49, *p* = 0.92, pre (white) and post placebo (dark blue): *n* = 8, pre (white) and post PDE9i (dark orange): *n* = 8 mice; Supplementary Figure 2D, Kruskal–Wallis test, H (3) = 3.47, *p* = 0.32, pre (white) and post placebo (light blue): *n* = 9, pre (white) and post PDE9i (light orange): *n* = 11 mice], suggesting that even profound differences in blood-corticosterone levels had no impact on cochlear mechanics.

In conclusion, in *high compensators* but not *low compensators*, a significant elevation of blood-corticosterone levels is induced by 10 consecutive days of injection with either the placebo or PDE9i. This suggests that *low compensators* exhibit a blunted stress response. Neither the variation in the corticosterone level seen between *high* and *low compensators* nor the placebo or PDE9i treatment had any influence on hearing thresholds.

### *High Compensators*, but Not *Low Compensators*, Exhibit a “Stress-Induced” Drop in Auditory Nerve Response That Can Be Restored by PDE9i Treatment

**Figure 4:**
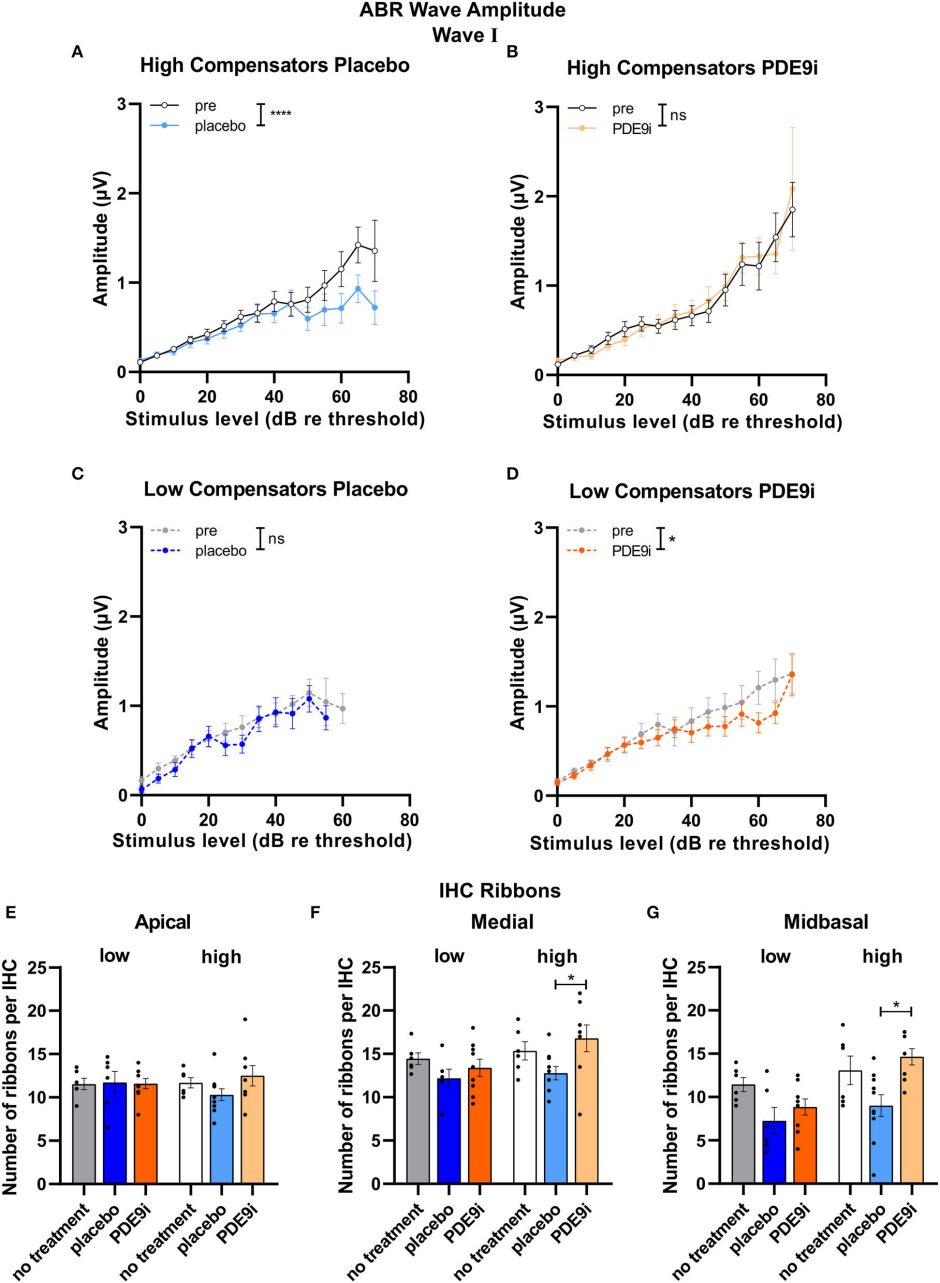
ABR wave I and IHC ribbon numbers in high and low compensators before and after treatment with placebo or PDE9i. **(A)** High compensators showed a significant decrease in ABR wave I amplitude after treatment with placebo. **(B)** However, in high compensators treated with PDE9i, this decrease was not present. Low compensators showed no change in ABR wave I amplitude after treatment with **(C)** placebo **(D)** and a significantly decreased ABR wave I amplitude after treatment with PDE9i. **(E)** IHC ribbon number in the apical cochlear turn, representing lower-frequency areas, shows no changes between the groups. **(F)** IHC ribbon number in the medial cochlear turn, representing higher-frequency areas, significantly less IHC ribbons were observed in high compensators treated with placebo in comparison to those treated with PDE9i. **(G)** In the midbasal cochlear turn, representing high-frequency areas, a significantly lower IHC ribbon number was found in high compensators treated with placebo in comparison to those treated with PDE9i. Mean ± SEM. ns *p* > 0.1, *p < 0.05, ****p < 0.0001. For **(A–D)**, results of repeated measures 2-way ANOVA and for **(E–G)** results of the Sidak's multiple comparison test are depicted.

We next asked whether suprathreshold auditory processing differences might exist independently of the hearing threshold between *low* and *high compensators* that are possibly related to the differences in corticosterone levels. We studied suprathreshold click-ABR wave fine structures and the temporal coding properties (ASSR) in both groups in more detail. Strikingly, in *high compensators* treated with a placebo, the amplitude of ABR wave I showed a significant decrease after treatment [Figure 4A, light blue line, repeated measures 2-way ANOVA, *F*(1, 138) = 4.92, *p* = 0.03, Sidak's multiple comparisons test, *n* = 20/10 ears/mice]. However, in *high compensators* treated with PDE9i, the wave I amplitude was preserved [Figure 4B, light orange line, repeated measures 2-way ANOVA, *F*(1, 124) = 0.15, *p* = 0.70, *n* = 22/11 ears/mice]. Similar results were observed in ABR wave IV, where *high compensators* showed a decreased amplitude after treatment with placebo, particularly at higher sound pressure levels [Supplementary Figure 3A, repeated measures 2-way ANOVA, *F*(1, 123) = 5.72, *p* = 0.02, *n* = 20/10 ears/mice]. After PDE9i treatment, *high compensators* showed no significant change in wave IV amplitude relative to the baseline [Supplementary Figure 3B, repeated measures 2-way ANOVA, *F*(1, 127) = 2.49, *p* = 0.12, *n* = 22/11 ears/mice], suggesting that PDE9i treatment continues to preserve auditory responses. In contrast to the change in auditory nerve responses to injection stress in *high compensators, low compensators* showed no differences in ABR wave I amplitude after placebo [Figure 4C, the dark blue line, repeated measures 2-way ANOVA, *F*(1, 44) = 2.04, *p* = 0.16, *n* = 8/4 ears/mice]; however, after PDE9i treatment, a significant reduction in ABR wave I amplitude was observed [Figure 4D, dark orange line, repeated measures, 2-way ANOVA, *F*(1, 78) = 4.90, *p* =0.03, *n* = 12/6 ears/mice]. ABR wave IV amplitudes were not affected by either PDE9i or placebo treatment in *low compensators* [Supplementary Figure 3C, repeated measures 2-way ANOVA, *F*(1, 47) = 0.06, *p* = 0.82, *n* = 8/4 ears/mice; Supplementary Figure 3D, repeated measures 2-way ANOVA, *F*(1, 77) = 2.27, *p* = 0.14, *n* = 12/6 ears/mice].

As a possible rationale for the observed differences in auditory processing between *low* and *high compensators* in response to injection-induced stress, we considered differences in the number of IHC ribbons. To estimate the level of deafferentation, we quantified the number of CtBP2/RIBEYE-immunopositive dots as indicators for ribbon synapses, opposing neurofilament-200 positive auditory fibers of middle-aged, untreated, placebo-, or PDE9i-treated animals.

We observed no differences in number of CtBP2/RIBEYE-immunopositive dots between the groups in the apical turn [Figure 4E, two-tailed Student's t-test: *t*(10) = 0.16, *p* = 0.88, low no treatment: *n* = 6, high no treatment: *n* = 6 mice; 1-way ANOVA, *F*(2, 18) = 0.01, *p* = 0.99, low no treatment: *n* = 6, low placebo: *n* = 6, low PDE9i: *n* = 9; 1-way ANOVA, *F*(2, 21) = 1.80, *p* = 0.19, high no treatment: *n* = 6, high placebo: *n* = 10, high PDE9i: *n* = 8 mice]. The number of CtBP2/RIBEYE-immunopositive dots in the medial turn of the cochlea, representing regions coding lower-frequency sounds, was not different between untreated *low* and *high compensators* [Figure 4F, two-tailed Student's t-test: *t*(10) = 0.73, *p* = 0.48, low no treatment: *n* = 6, high no treatment: *n* = 6 mice], nor were any significant differences observed in *low compensators* after either treatment [Figure 4F, 1-way ANOVA, *F*(2, 18) = 1.19, *p* = 0.33, low no treatment: *n* = 6, low placebo: *n* = 6, low PDE9i: *n* = 9]; however, *high compensators* treated with placebo had significantly lower IHC ribbon numbers in comparison to *high compensators* treated with PDE9i [Figure 4F, 1-way ANOVA, *F*(2, 21) = 3.54, *p* = 0.048, Sidak's multiple comparisons test, high no treatment: *n* = 6, high placebo: *n* = 10, high PDE9i: *n* = 8 mice]. In the midbasal cochlear turn, which codes higher frequencies, untreated *low* and *high compensators* did not differ in IHC ribbon number [Figure 4G, two-tailed Student's t-test: *t*(10) = 0.89, *p* = 0.40, low no treatment: *n* = 6, high no treatment: *n* = 6 mice]. In addition, despite a trending group effect, no significant differences were observed in *low compensators* after treatment with either placebo or PDE9i [Figure 4G, 1-way ANOVA, *F*(2, 18) = 3.15, *p* = 0.07, low no treatment: *n* = 6, low placebo: *n* = 6, low PDE9i: *n* = 9]; however, *high compensators* treated with placebo had significantly lower IHC ribbon numbers in comparison to those treated with PDE9i [Figure 4F, 1-way ANOVA, *F*(2, 21) = 5.78, *p* = 0.01, Sidak's multiple comparisons test, high no treatment: *n* = 6, high placebo: *n* = 10, high PDE9i: *n* = 8 mice], reflecting ABR wave I amplitude results.

**Figure 5:**
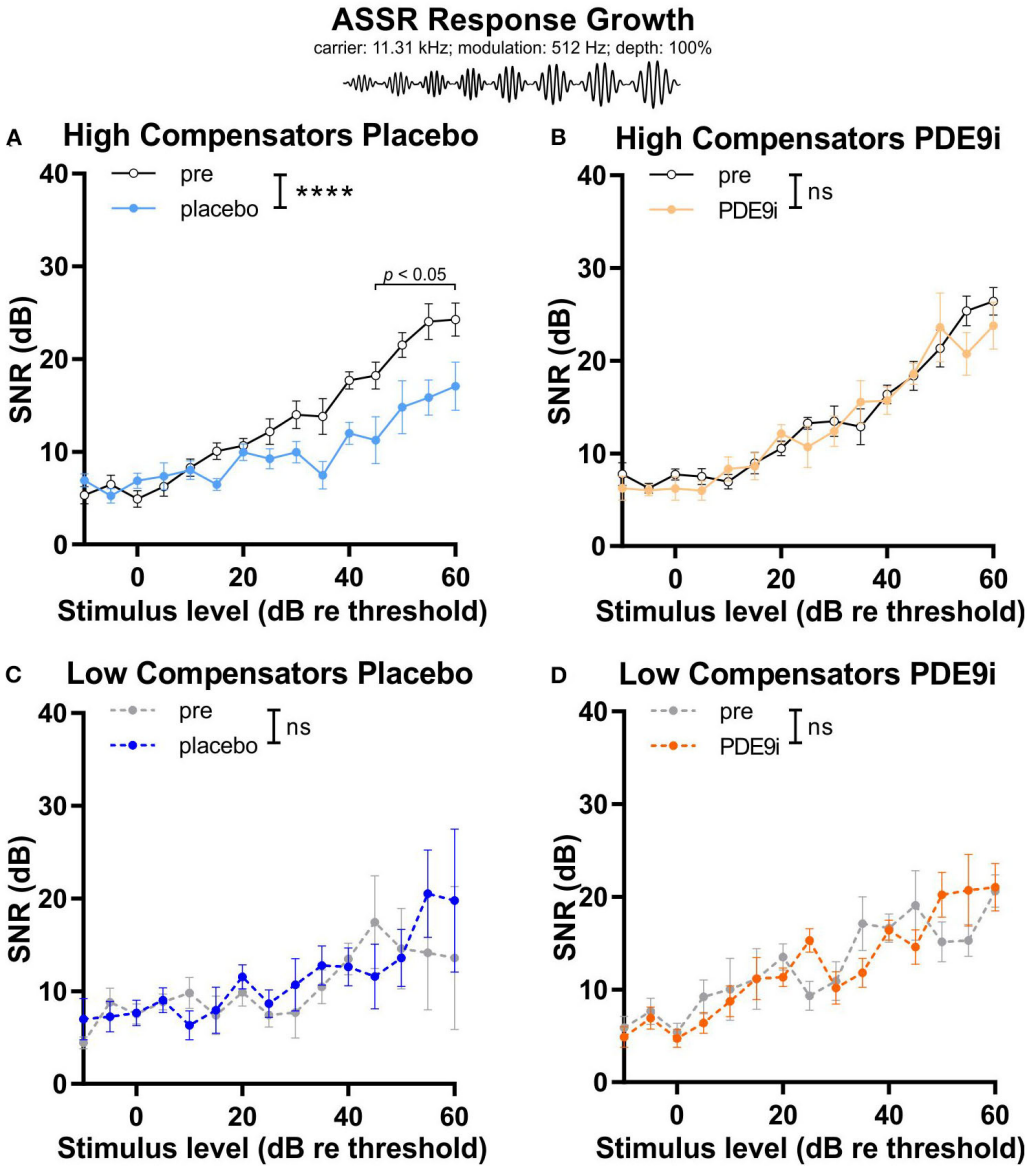
Input–output relationship of auditory steady state responses (carrier frequency: 11.32 kHz; modulation frequency: 512 Hz; modulation depth: 100%) in high and low compensators treated with either placebo or PDE9i. **(A)** High compensators also showed a significant decrease of temporal auditory resolution after treatment with placebo. **(B)** However, in high compensators treated with PDE9i, this decrease was not present. Low compensators showed no change in temporal auditory resolution after treatment with either **(C)** placebo or **(D)** PDE9i. Mean ± SEM. ns *p* > 0.1, ****p < 0.0001, repeated measures 2-way ANOVA.

A similar differential effect in response to placebo or PDE9i treatment between *high* and *low compensators* was observed when we measured the ASSR growth function in response to increasing sound pressure levels. In *high compensators*, a significant decrease of SNR of the ASSR growth function was observed after placebo treatment [Figure 5A, light blue line, repeated measures 2-way ANOVA, *F*(1, 122) = 44.30, *p* < 0.0001, Sidak's multiple comparisons test, *n* = 10 mice], a feature that could be prevented by PDE9i treatment [Figure 5B, light orange line, repeated measures 2-way ANOVA, *F*(1, 106) = 1.52, *p* = 0.22, *n* = 10 mice]. In contrast, in *low compensators*, the temporal resolution was not affected by either placebo [Figure 5C, the dark-blue line, repeated measures, 2-way ANOVA, *F*(1, 56) = 1.05, *p* = 0.31, *n* = 6 mice] or PDE9i treatment [Figure 5D, dark orange line, repeated measures 2-way ANOVA, *F*(1, 86) = 0.01, *p* = 0.94, *n* = 8 mice].

This indicates that, in *high compensators*, a glucocorticoid (GC)-sensitive and cyclic guanosine-monophosphate (cGMP)-sensitive component is maintained, and that this component contributes to synchronous auditory nerve responses and temporal coding and could possibly be impaired in *low compensators*.

In conclusion, these results suggest that the stressful placebo injection disrupts discharge rates and/or synchronous auditory nerve responses, as well as temporal coding in *high compensators*, a feature that can be counteracted by PDE9i treatment. *Low compensators*, in contrast, have lost their capacity to respond to stressful events and, therefore, do not profit from treatment with PDE9i.

### *High Compensators*, but Not *Low Compensators*, Exhibit a “Stress-Induced” Drop in Hippocampal LTP That Can Be Preserved by PDE9i Treatment

**Figure 6:**
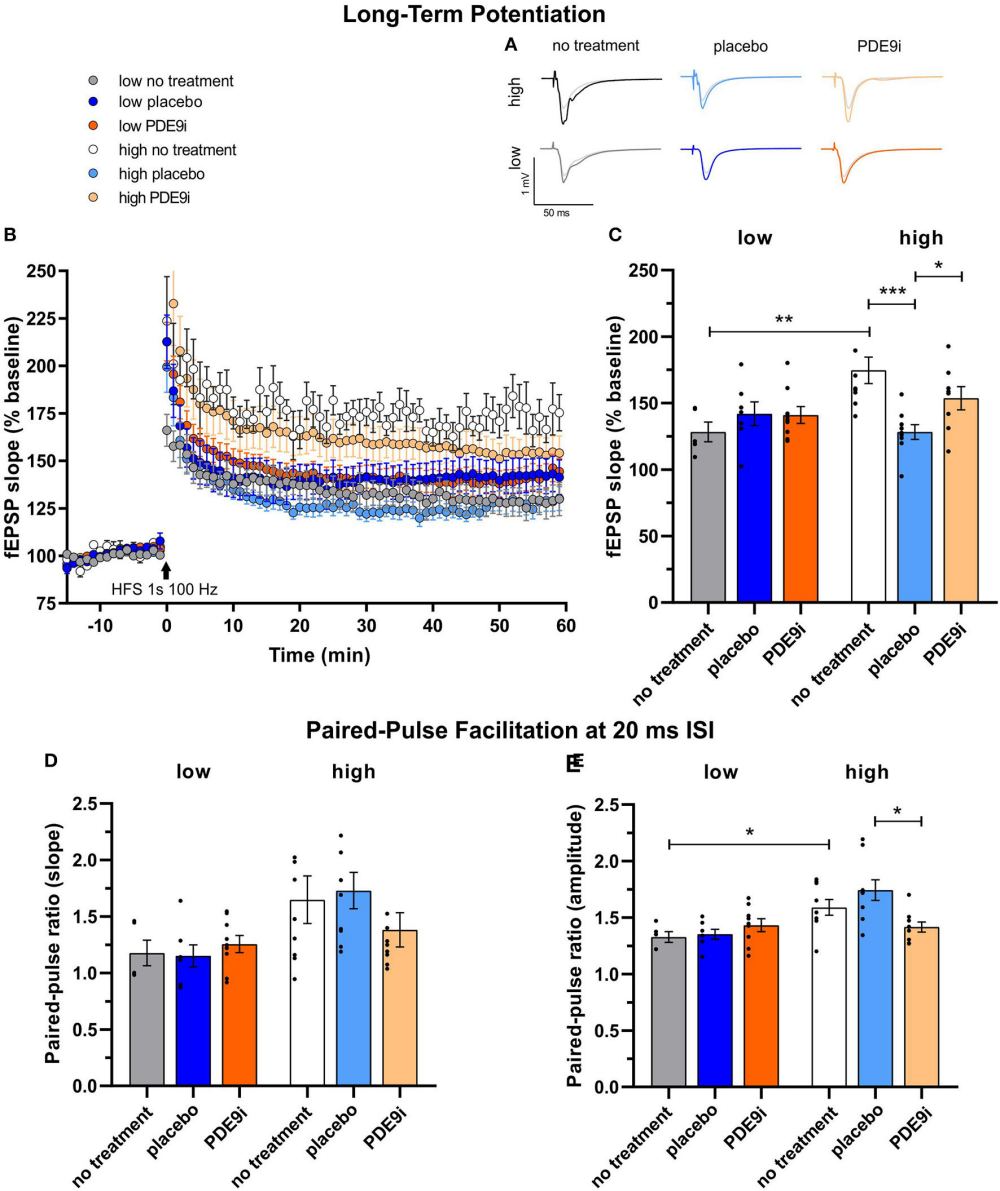
Long-term potentiation (LTP) and paired-pulse facilitation (PPF) of low and high compensators that underwent no treatment, placebo treatment, or PDE9i treatment. **(A)** Representative traces before and after LTP induction and **(B)** averaged time courses of fEPSP slopes in acute coronal brain slices displayed prominent LTP in all animal groups. **(C)** The untreated low compensators had significantly lower LTP in comparison to untreated high compensators. In low compensators, neither a placebo treatment nor PDE9i treatment showed any effect on their already low LTP. In contrast, high compensators treated with a placebo displayed a significantly lower LTP both in comparison to their untreated controls and to high compensators treated with PDE9i. **(D)** The analysis of the paired-pulse ratio of the fEPSP2/fEPSP1 slope at the 20 ms ISI showed no significant differences between high and low compensators before treatment. Neither low compensators nor high compensators treated with either a placebo or PDE9i differed from their untreated controls. Also, no difference in the PPF slope ratio (20 ms ISI) between the treatment conditions of low and high compensators was observed. **(E)** The analysis of the ratio of the paired-pulse ratio of the fEPSP2/fEPSP1 amplitude at the 20 ms interstimulus interval revealed a significantly higher PPF amplitude ratio in untreated high compensators in comparison to untreated low compensators. High compensators treated with placebo had a significantly higher PPF amplitude ratio (ISI 20 ms) in comparison to those treated with PDE9i, but neither placebo- nor PDE9i-treated high compensators significantly differed from their untreated controls. By contrast, low compensators showed no differences after treatment with either a placebo or PDE9i as compared to untreated controls or compared with each other. Mean ± SEM.*p < 0.05, **p < 0.01, ***p < 0.001. For the comparison of the untreated groups in **(C,E)**, results of the two-tailed t-test are depicted. For the comparison of the differentially treated high compensators in **(C,E)**, results of the Sidak's multiple comparison test are depicted.

Stress and noise exposure have been shown to impact cognitive functions, including LTP (Singer et al., 2013; Jafari et al., 2018; Matt et al., 2018; Zhang et al., 2022), motivating us to compare LTP between treated and untreated *high* and *low compensators*. LTP recordings were conducted on acute coronal brain slices of mice without any treatment or after completion of treatment with a placebo or PDE9i (Figure 6A, averaged time course). LTP was induced by tetanic stimulation (1 s, 100 Hz) to the CA3 Schaffer's collateral axons, and fEPSPs were recorded from the dendrites of CA1 pyramidal cells that form synaptic contacts with CA3 Schaffer's collateral axons (Matt et al., 2018). The mean of the last 10 min of the 60 min recording was averaged and compared [Figure 6B, 1-way ANOVA, *F*(6, 49) = 11.17, *p* < 0.0001]. The post high-frequency stimulation (HFS) fEPSPs of the untreated mice were significantly higher in *high compensators* (Figures 6B,C, white, 174.69% ± 9.96%; n = 4/9 animals/slices) in comparison to *low compensators* [Figure 6B, gray, 128.35% ± 7.46%, *n* = 2/5 animals/slices; Figure 6C, two-tailed Student's t-test: *t* (12) = 3.17, *p* = 0.008], reminiscent of the higher corticosterone levels in *high compensators* in comparison to *low compensators* (Figure 3).

*Low compensators* treated either with placebo (Figures 6B,C, dark blue, 142% ± 9%, *n* = 2/7 animals/slices) or PDE9i (Figures 6B,C, dark orange, 141% ± 6.2%, *n* = 3/9 animals/slices) showed no significant change in LTP in comparison to the untreated group [Figures 6B,C, gray, 1-way ANOVA, *F*(2, 18) = 0.81, *p* = 0.46]. In contrast, in the *high compensators*, placebo treatment caused a significant reduction in the LTP level compared to the untreated mice [Figure 6B, light blue, 128.3% ± 5.5%, *n* = 3/9 animals/slices; Figure 6C, 1-way ANOVA, *F*(2, 23) = 8.24, *p* = 0.002, with a two-stage linear step-up procedure of Benjamini, Krieger, and Yekutieli]. Treatment with PDE9i (Figures 6B,C, light orange, 153% ± 8.6%, *n* = 2/8 animals/slices) prevented the reduction in LTP caused by the placebo injection, shifting it to a level significantly higher than in the placebo-treated group.

Importantly, basic synaptic transmission is not impaired in *high* or *low compensators* (Supplementary Figure 4). Representative traces of fEPSPs with increasing stimulus intensities from 25 μA to 150 μA in 25 μA steps are shown in Supplementary Figure 4A. After either placebo or PDE9i treatment in both *high* and *low compensators*, the fEPSP slope and fiber volley (FV) amplitude was increased [Supplementary Figure 4B, high compensators: 2-way ANOVA, *F*(2, 144) = 10.97, *p* < 0.0001, a two-stage linear step-up procedure of Benjamini, Krieger, and Yekutieli, high no treatment: *n* = 4/9, high placebo: *n* = 3/9, high PDE9i: *n* = 2/8 animals/slices, low compensators: 2-way ANOVA, *F*(2, 108) = 17.72 *p* < 0.0001, a two-stage linear step-up procedure of Benjamini, Krieger, and Yekutieli, low no treatment: *n* = 2/5, low placebo: *n* = 2/7, low PDE9i: *n* = 3/9 animals/slices; Supplementary Figure 4C, high compensators: 2-way ANOVA, *F*(2, 144) = 59.17, *p* < 0.0001, a two-stage linear step-up procedure of Benjamini, Krieger, and Yekutieli, high no treatment: *n* = 4/9, high placebo: *n* = 3/9, high PDE9i: *n* = 2/8 animals/slices, low compensators: 2-way ANOVA, *F*(2, 108) = 7.71, *p* = 0.0007, a two-stage linear step-up procedure of Benjamini, Krieger, and Yekutieli, low no treatment: *n* = 2/5, low placebo: *n* = 2/7, low PDE9i: *n* = 3/9 animals/slices]. The fEPSP slope and FV amplitudes were, however, increased at a level that was proportional to one another [Supplementary Figure 4D, high compensators: simple linear regression analysis, comparison between slopes of lines, *F*(2, 155) = 0.45, *p* = 0.64, high no treatment: *n* = 4/9, high placebo: *n* = 3/9, high PDE9i: *n* = 2/8 animals/slices, low compensators: simple linear regression analysis, comparison between slopes of lines, *F*(2, 120) = 0.04, *p* = 0.96, low no treatment: *n* = 2/5, low placebo: *n* = 2/7, low PDE9i: *n* = 3/9 animals/slices]. This suggests that the signal transmission was intact in all treatment conditions and compensation groups but operates with a lower ratio at a higher set point after treatment with a placebo or PDE9i. This suggests that the changes in LTP were not due to changes in basic synaptic transmission.

To investigate the effect of compensation group or treatment mechanism on the presynaptic state, we studied paired-pulse facilitation (PPF), a simple form of presynaptic short-term plasticity, which has been explained as a transient increase in the probability of vesicular glutamate release (Satake et al., 2012).

We had studied the time course of PPF in each brain slice before LTP induction in animals that underwent no treatment, placebo injection, or PDE9i treatment using varying interstimulus intervals (ISIs, 10–20–50–100–200–500 ms) and the same stimulation strength used for LTP recordings (Figures 6D,E, Supplementary Figure 5). Representative traces of fEPSPs with increasing ISIs at the stimulation strength equal to corresponding LTP recordings are shown in Supplementary Figure 5A. Specifically, *low compensators* had a significantly lower PPF amplitude ratio for the 20 ms ISI in comparison to *high compensators* [Figure 6E, gray vs. white, two-tailed Student's t-test: *t*(12) = 2.58, *p* = 0.02, low no treatment: *n* = 2/5, high no treatment: *n* = 4/9 animals/slices]. In *high compensators*, the PPF amplitude ratio for ISIs up to 20 ms could be even more enhanced by placebo treatment, resulting in a significant increase in comparison to their untreated controls [Supplementary Figure 5B, light blue vs. white, 2-way ANOVA, *F*(2, 144) = 9.07, *p* = 0.0002, a two-stage linear step-up procedure of Benjamini, Krieger, and Yekutieli, high no treatment: *n* = 4/9, high placebo: *n* = 3/9, high PDE9i: *n* = 2/8 animals/slices; Supplementary Figure 5C, light blue vs. white, 2-way ANOVA, *F*(2, 144) = 24.04, *p* < 0.0001, a two-stage linear step-up procedure of Benjamini, Krieger, and Yekutieli, high no treatment: *n* = 4/9, high placebo: *n* = 3/9, high PDE9i: *n* = 2/8 animals/slices], while PDE9i significantly decreased the PPF amplitude and the slope ratio for ISIs up to 20 ms in comparison to untreated controls (Supplementary Figures 5B,C, light orange vs. white). Significant effects on the PPF slope [Supplementary Figure 5D, 2-way ANOVA, *F*(2, 108) = 4.40, *p* = 0.02] and the amplitude [Supplementary Figure 5E, 2-way ANOVA, *F*(2, 108) = 6.09, *p* = 0.003] ratio were also observed for *low compensators*. In contrast to *high compensators, low compensators* showed a significant increase in the PPF amplitude ratio only at the 10 ms ISI both after placebo [Supplementary Figure 5E, dark blue, 2-way ANOVA, *F*(2, 108) = 6.09, *p* = 0.003, a two-stage linear step-up procedure of Benjamini, Krieger, and Yekutieli, low no treatment: *n* = 2/5, low placebo: *n* = 2/7, low PDE9i: *n* = 3/9 animals/slices] and PDE9i treatment (Supplementary Figure 5E, dark orange) in comparison to their untreated controls (Supplementary Figure 5E, gray). Also, in *low compensators*, no difference between placebo and PDE9i treatment conditions was indicated (Figures 6D,E, Supplementary Figure 5E).

This suggests that a transient increase in the probability of vesicular glutamate release tends to be higher in *high compensators* than in *low compensators*. Only in *high compensators* this feature can be enhanced through injection-induced stress and subsequently lowered through PDE9i treatment. This may suggest that, in *high compensators*, but not *low compensators*, stress-induced facilitated presynaptic release (Figures 6D,E, light blue) contributes to the retention of LTP (Figure 6C, light blue).

In conclusion, this finding suggests that *low compensators* with a blunted stress response have lower hippocampal LTP and PPF than *high compensators*. As placebo treatment can reduce and PDE9i treatment can restore LTP in *high compensators*, a GC- and cGMP-signaling-dependent facilitation circuit exists in this animal group that is impaired in *low compensators*.

### *High Compensators*, but Not *Low Compensators*, Exhibit a “Stress-Induced” Drop in Levels of Hippocampal BDNF and Inhibitory Markers That Can Be Preserved by PDE9i Treatment

**Figure 7:**
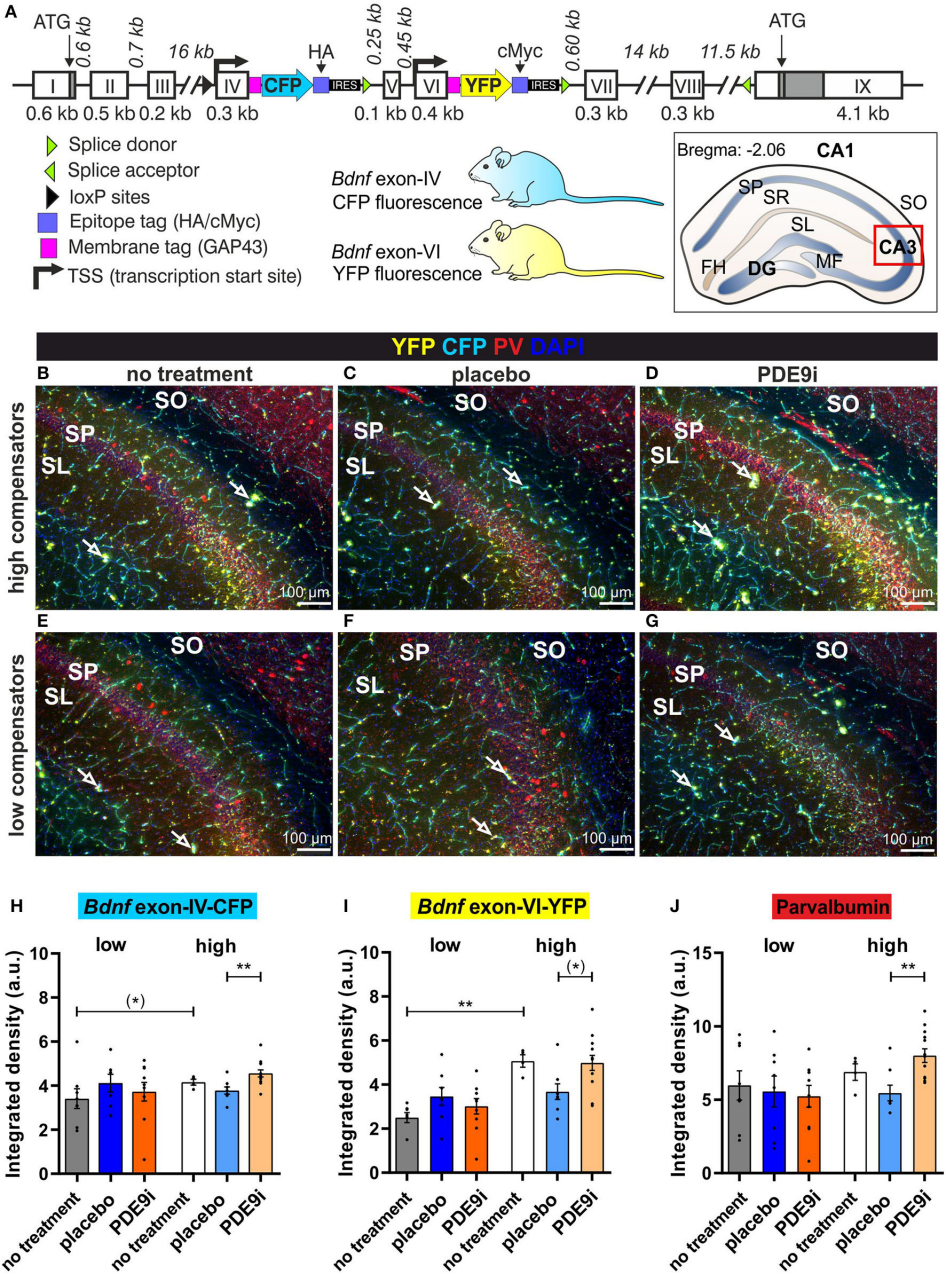
*Bdnf* exon-IV, *Bdnf* exon-VI, and Parvalbumin (PV) expression in the CA3 region of the hippocampus of untreated low and high compensators and animals that received placebo or PDE9i. **(A)** Genetically modified the *Bdnf* gene of the BLEV reporter mouse line in which CFP is expressed with activity-dependent *Bdnf* exon-IV transcription and YFP with activity-dependent *Bdnf* exon-VI transcription. An abstract scheme of a coronal hippocampal section at bregma position −2.06 mm. The red box indicates the inset seen in **(B–G)** where the integrated density is measured. DG, dentate gyrus; FH, fissura hippocampalis; MF, mossy fiber; SL, stratum lucidum; SO, stratum oriens; SP, stratum pyramidale; SR, stratum radiatum. **(B)** Untreated high compensators showed a strong expression of *Bdnf* exon-IV (CFP), *Bdnf* exon-VI (YFP) (open arrows signify where they are expressed in neighboring structures) and PV (red) in the hippocampal CA3 region (blue = DAPI). **(C)** After placebo treatment, less expression of CFP, YFP, and PV was observed. **(D)** When treated with PDE9i, high compensators showed an increase in CFP, YFP, and PV expression. **(E–G)** CFP, YFP, and PV expression in the hippocampal CA3 region of low compensators, which was slightly weaker than in high compensators, reaching significance for YFP expression in untreated animals, shown in **(I)**. In low compensators, neither placebo nor PDE9i treatment had an effect on CFP, YFP, and PV expression. **(H)** In the SL/SP region of CA3, untreated low compensators showed a lower CFP expression in comparison to untreated high compensators not reaching statistical significance (*). Low compensators treated with placebo or PDE9i did not show any differences in CFP expression to their untreated controls or to each other. By contrast, high compensators treated with PDE9i had significantly higher CFP expression in comparison to those treated with placebo. **(I)** YFP expression in the SL/SP region of CA3 was significantly lower in untreated low compensators in comparison to untreated high compensators. Low compensators showed no difference in YFP expression between treatment groups. However, high compensators treated with PDE9i showed a higher YFP expression in comparison to those treated with placebo not reaching statistical significance. **(J)** PV expression in the SL/SP region of CA3 did not differ between untreated high and low compensators. Low compensators showed no difference in PV expression between treatment groups. High compensators treated with PDE9i showed a significantly higher PV expression compared to those treated with placebo. Mean ± SEM. (*)*p* ≤ 0.1, **p < 0.01. For the comparison of the untreated groups in **(H–J)**, results of two-tailed t-test are depicted. For the comparison of the differentially treated high compensators in **(H–J)**, results of the Dunn's multiple comparison test are depicted.

We next investigated the influence of injection-induced stress or application of PDE9i on activity-dependent changes in *Bdnf* exon-IV and -VI expression, tagged by bi-cistronic expression of CFP or YFP, respectively, monitored in the BLEV reporter mice (Figure 7A). We examined *Bdnf* exon-IV-CFP and exon-VI-YFP expression (Figures 7B–G, cyan and yellow) as well as parvalbumin (PV) protein staining (Figures 7B–G, red) in deconvoluted high-resolution fluorescence stacks in the hippocampus (a framed schematic view in Figures 7A–G) (Matt et al., 2018).

We found that *low compensators* had a lower expression level of *Bdnf* exon-IV-CFP and a lower *Bdnf* exon-VI-YFP in CA3 regions of the dorsal hippocampus in comparison to *high compensators*, not always reaching significance [Figure 7H, gray vs. white bar, Mann–Whitney U test, U(3.255, 4.197) = 5; *p* = 0.07, low: *n* = 5/8, high: *n* = 2/4 animals/hippocampal hemispheres; Figure 7I, gray vs. white bar, Mann–Whitney U test, U(2.749, 5.218) = 0; *p* = 0.004, low: *n* = 5/8, high: *n* = 2/4 animals/hippocampal hemispheres], but their PV expression used to identify changes in fast-spiking GABAergic interneurons (Hu et al., 2014) did not differ [Figure 7J, gray vs. white bar, Mann–Whitney U test, U(5.550, 7.208) = 13, *p* = 0.68, low: *n* = 5/8, high: *n* = 2/4 animals/hippocampal hemispheres]. In *low compensators*, treatment with neither placebo nor PDE9i significantly affected the expression of *Bdnf* exon-IV-CFP, *Bdnf* exon-VI-YFP, or PV [Figure 7H, gray vs. dark blue and dark orange bars, Kruskal–Wallis test, H (2) = 1.71, *p* = 0.43, low no treatment: *n* = 5/8, low placebo: *n* = 5/8, low PDE9i: *n* = 4/7 animals/hippocampal hemispheres; Figure 7I, gray vs. dark blue and dark orange bars, Kruskal–Wallis test, H (2) = 4.83, *p* = 0.09, low no treatment: *n* = 5/8, low placebo: *n* = 5/8, low PDE9i: *n* = 4/8 animals/hippocampal hemispheres; Figure 7J, gray vs. dark blue and dark orange bars, Kruskal–Wallis test, H (2) = 0.25, *p* = 0.88, low no treatment: *n* = 5/8, low placebo: *n* = 4/8, low PDE9i: *n* = 4/8 animals/hippocampal hemispheres]. However, in *high compensators*, PDE9i treatment was able to increase the expression of *Bdnf* exon-IV-CFP, *Bdnf* exon-VI-YFP, and PV, in comparison to the placebo treatment where it was slightly diminished [Figure 7H, light orange bars vs. light blue bars, Kruskal–Wallis test, H (2) = 9.42, *p* = 0.009, Dunn's multiple comparisons test, high no treatment: *n* = 2/4, high placebo: *n* = 2/4, high PDE9i: *n* = 4/8 animals/hippocampal hemispheres; Figure 7I, light orange bars vs. light blue bars, Kruskal–Wallis test, H (2) = 6.05, *p* = 0.049, Dunn's multiple comparisons test, high no treatment: *n* = 2/4, high placebo: *n* = 2/4, high PDE9i: *n* = 4/8 animals/hippocampal hemispheres; Figure 7J, light orange bars vs. light blue bars, Kruskal–Wallis test, H (2) = 8.82, *p* = 0.01, Dunn's multiple comparisons test, high no treatment: *n* = 2/4, high placebo: *n* = 2/4, high PDE9i: *n* = 4/8 animals/hippocampal hemispheres]. A significant difference between treatment and untreated controls was not observed (Figure 7H, light orange bars/light blue bars vs. white bars).

In conclusion, treatment with a PDE9i can preserve a stress-induced decrease in expression levels of *Bdnf* exon-IV-CFP, *Bdnf* exon-VI-YFP, and PV in hippocampal regions in *high compensators*, but not *low compensators*.

## Discussion

The findings of the present study indicate for the first time that central neural responses (neural gain) to age-related cochlear synaptopathy may differ, depending on individual stress responses. Acute high stress induction can reduce increased cochlear and central auditory responses, including hippocampal LTP following cochlear synaptopathy. This stress-induced decrease in central adaptation capacity can be restored by elevating cGMP levels with a PDE9i. However, in case of blunted stress responses, both central neural gain and PDE9i responsiveness fail. This suggests that central neuronal enhancement needed for regular adaptation processes to sensory deprivation depends on proper stress responses and cGMP signaling.

### Auditory (Temporal) Processing Deficits in Middle-Aged Animals Differ Between *Low* and *High Compensators* Independent of Hearing Thresholds

In the present study, we confirmed a previously made observation that animals can respond to an age-dependent reduction in auditory nerve amplitude (cochlear synaptopathy) in two different ways: through either lower or higher compensations in the central ABR wave IV responses (Marchetta et al., 2020b). Although during aging, the animals showed a homogenous elevation in hearing threshold (Figure 1), the middle-aged animals could be subdivided into two groups based on a better (*high compensators*) or worse (*low compensators*) ability to centrally compensate for the peripheral damage. Between these newly defined groups generated for the present study, hearing thresholds did not differ in response to click, noise, or pure-tone 11 kHz stimuli (Figures 2A–C). However, threshold sensitivity in response to pure-tone stimuli over a frequency range of 2–32 kHz was significantly worse in *low compensators* in comparison to *high compensators* in the best frequency range (Figure 2D). In addition, temporal auditory resolution was also significantly lower (Figure 2E). While this finding confirmed previous observations (Marchetta et al., 2020b), here, we found that the weaker sound responsiveness and lower temporal precision of *low compensators* were related to a significantly lower baseline corticosterone level in comparison to *high compensators* (Figure 3). Furthermore, *low compensators* did not respond to injection-induced stress (Figure 3), while, in *high compensators*, peripheral and central auditory responsiveness and temporal acuity were reduced by injection-induced stress (Figures 4, 5). Importantly, the differences in corticosterone levels in both groups induced by injection stress did not influence hearing thresholds (Supplementary Figure 2).

We hypothesize that this finding discloses two important new features of auditory processing: (i) There is a top-down influence of GCs on peripheral auditory processing, independent of any impact on the electromechanical properties of OHCs, and (ii) auditory processing can profoundly differ depending on the extra-hypothalamic stress control [i.e., modulation through the prefrontal cortex (PFC) or hippocampal regions (de Kloet et al., 2005, 2019)] of the individual specimen. Central auditory responses can either fall into a group of *low compensators* with characteristics of a so-called blunted or low stress reactivity, possibly being in a state of chronic stress (Turner et al., 2020), or a group of *high compensators* that can maintain stable neuronal activity in central auditory circuits despite auditory deprivation but which risk losing it under acute high-stress conditions.

(i) In line with the present study, a top-down influence of differences in stress responses, independent of hearing thresholds and thus electro mechanical properties of OHCs, has previously been shown. Thus, maintained high levels of endogenous corticosterone or glucocorticoid receptor (GR) antagonists in acoustic trauma did not influence hearing thresholds or the distortion product of otoacoustic emissions in rats (Singer et al., 2018a). Furthermore, posttraumatic or chronic stress has not yet been shown to directly influence hearing thresholds in humans or animals (Kreuzer et al., 2014; Mazurek et al., 2019; Turner et al., 2019; MacGregor et al., 2020), indicating that stressors may influence neuronal rather than mechanical properties of hair cells. When asking how stress could possibly influence neuronal cochlear processes, one should remember that stressors act through GR and mineralocorticoid receptors (MR) in the limbic system, including the PFC and hippocampus, and are suggested to mediate the top-down and bottom-up control of stress, coping with environmental challenges through hypothalamic and extra-hypothalamic pathways (de Kloet et al., 2000, 2019). In a study that performed tamoxifen-induced single or double deletion of MR and GR in the central nervous system of mice, an influence on cochlear sensitivity, as measured by ABR wave I, IHC ribbon number, a compound action-potential threshold, and neural temporal sound processing (ASSR) was shown to occur independently of changes in the mechanics of cochlear OHCs, as measured *via* the distortion-product otoacoustic emissions (Marchetta et al., 2022). This indicates that central stress receptor activation may directly affect cochlear processes.
(ii) Considering that stress receptor activation could modify the neural properties of cochlear auditory processing, higher endogenous corticosterone levels were shown to lower the summed response amplitudes of the auditory nerve and were linked to fewer IHC ribbons in the midbasal cochlear turn of rats (Singer et al., 2018a). Furthermore, an acoustic trauma-induced attenuation of ABR wave I amplitude and diminution of its dynamic range could be counteracted and partially restored by the GR antagonist mifepristone (Singer et al., 2018a). Moreover, differences in auditory nerve processing could be observed, following a deletion of either MR or GR in frontal brain regions (Marchetta et al., 2022). In that study, we deleted MR and GR through a tamoxifen**-**induced, calmodulin-dependent protein kinase II alpha promoter activation, what restricts the MR/GR deletion to central and frontal brain regions, while leaving MR and GR expression in the cochlea intact (Marchetta et al., 2022). As a consequence, the deletion of MR in the frontal brain regions of the adult mice led to impaired peripheral auditory processing shown by a reduced number of IHC ribbons and ABR wave I amplitudes. This might point to a positive retro-cochlear feedback that is dependent on central MR activation (Marchetta et al., 2022) to preserve auditory processing. As *low compensators* show an overall phenotype of poorer auditory processing and have a low baseline corticosterone level, we may hypothesize that they have an inappropriate MR activation. Additionally, *low compensators* might also have an impaired GR response, in contrast to *high compensators* (Figure 3), because also prolonged stress exposure–in this study, 10 days of repeated injections–did not lead to increased corticosterone levels.

While MRs show a high affinity for endogenous GC, which makes them responsive during acute stress events (de Kloet et al., 2005; Joels and de Kloet, 2017; Wirz et al., 2017; Plieger et al., 2018), GRs are typically activated in response to the highest GC level, since GRs, although more widely and extensively expressed, have only one tenth of the affinity for GCs in comparison to MR (de Kloet et al., 1999, 2005; Sapolsky, 2015). *Low compensators* exhibit diminished responsiveness to GCs and thereby the neurons in the auditory pathway would consequently be prevented from adequately responding to even low levels of GCs, which lead to the positive effects of MR (Marchetta et al., 2022). In humans and rodents with a likewise GC resistance or low and “blunted” stress response, endogenous GCs fail to induce proper responses to stress and circadian cues, which regulate life-sustaining processes, such as metabolism under stressful conditions (Lewis-Tuffin and Cidlowski, 2006; Silverman and Sternberg, 2008; Vitellius et al., 2018; Spies et al., 2021). In future studies, it may thus be interesting if more generalized defects, aside from lower auditory and cognitive performance, may be seen in *low compensators*. In contrast, *high compensators*, which respond to placebo-induced injection stress with a large increase in corticosterone levels (Figure 3), show a reduction in ABR wave IV amplitude and decrease in the ASSR after stress, as suggested after activation of central GR (Marchetta et al., 2022).

Future studies should, therefore, discuss central adaptation differences after cochlear synaptopathy in the context of different GR/MR-mediated feedback loops on cochlear processes, which may generally fail upon blunted stress levels.

### Short- and Long-Term Potentiation, *Bdnf* Transcript Recruitment, and PDE9i Sensitivity Differ Between *Low* and *High Compensators*

In the present study, *low* and *high compensators* differ in hippocampal LTP responses, as previously described (Marchetta et al., 2020b); here, we have linked this to differences in stress and cGMP signaling.

We suggest a strong relationship between hippocampal processes (i.e., LTP and *Bdnf* transcript levels) and auditory nerve responses, as they respond in the same way to stress and PDE9i-treatment. The *low compensators* are not only insensitive to a stress-induced deterioration of IHC ribbon numbers, ABR wave I amplitude, and ASSR but also to a stress-induced drop in hippocampal LTP (Figures 6B,C), to a recruitment of activity-dependent *Bdnf* transcripts (Figure 7), as well as to a stress-induced enhancement of the paired-pulse ratio (Figures 6D,E). Importantly, *low compensators* are also insensitive to the counteraction of all these effects by PDE9i.

In contrast to *low compensators, high compensators* respond to injection-induced stress not only through a drop of hippocampal LTP and *Bdnf* transcripts (Figure 7) but also with an enhanced PPF (Figures 6D,E), a simple form of pre-synaptically coded short-term plasticity (Neher and Sakaba, 2008). This suggests that, in *high compensators*, injection-induced stress may trigger a transient increase in the probability of vesicular release in Schaffer's collaterals, as previous studies have suggested that PPF results from a prior accumulation of residual Ca^2+^ at the synaptic terminal and a lingering effect of Ca^2+^ on the exocytotic Ca^2+^ sensor of releasable vesicles during the second stimulus [as reviewed in (Thomson, 2000; Zucker and Regehr, 2002)]. The stress-induced enhancement of PPF amplitude and the PPF slope ratio, observed here within the first stimulus intervals (< 100 ms) in *high compensators* (Supplementary Figures 4B,C), along with the reduced LTP response seen under the same stress conditions, points to a facilitation of vesicle release and a possible subsequent long-term depletion of releasable vesicles during trains of high-frequency presynaptic stimulation. A stress-induced facilitation of vesicle release may result from stress influences on the medio-dorsal thalamus (MD), which, along with the medial PFC, have been shown to interact with the hippocampal formation through topographically complex connectivity patterns (Bueno-Junior and Leite, 2018). Indeed, the inactivation of the MD through tetrodotoxin (Sloan et al., 2011) or chronic unpredictable stress (Jett et al., 2017) has been shown to significantly weaken MD/medial PFC recruitment (Jett et al., 2017), leading to LTP retention, as seen in the present study following injection-induced stress in *high compensators* (Figure 6). Importantly, in the present study, the corticosterone level in *high compensators* is increased independently of the treatment condition received (i.e., placebo or PDE9i, Figure 3). This indicates that the protective effects of the PDE9i in *high compensators* against the stress-induced diminishment of IHC ribbons, ABR wave I amplitude, ASSR, LTP, BDNF expression, and PPF may not be driven by hypothalamic-pituitary-adrenal (HPA) axis-induced changes in GC levels but, instead, downstream of it.

Consistent with the contrasting influences of stress on central auditory processes presented here in *high* and *low compensators*, acute stress or low corticosterone levels have been shown to exert positive influences on hearing (Meltser and Canlon, 2011; Canlon et al., 2013), while high corticosterone (i.e., posttraumatic or chronic stress) was shown to diminish auditory gating in animal and human studies (Stevens et al., 2001; White et al., 2005; Maxwell et al., 2006; Elling et al., 2011; Kreuzer et al., 2014; Ma et al., 2017; Mazurek et al., 2019; Turner et al., 2019; MacGregor et al., 2020). Moreover, MR activation was shown to exhibit a crucial role in proper neural responses of learning, memory, and selective attention to novel situations (de Kloet et al., 2005; Joels and de Kloet, 2017; Wirz et al., 2017; Plieger et al., 2018), while GR plays an important role in memory consolidation and long-term adaptation to stressful situations (de Kloet et al., 2005). This means that both stress receptors may play a central role in the learning-dependent adaptation processes after cochlear synaptopathy. The connection may lie in a differential influence of stress receptors on the recruitment of activity-dependent *Bdnf* transcripts necessary for adaptation, whose mobilization in *low* and *high compensators* is differentially affected by stress. BDNF has been suggested to bridge GC effects and brain networks by driving the phosphorylation of GRs (Jeanneteau et al., 2018). Impaired GR phosphorylation following a reduced activity-dependent BDNF recruitment has, moreover, been shown to lead to impaired long-term memory acquisition and deficits in forming postsynaptic dendritic spines after, for example, motor-skill training (Arango-Lievano et al., 2019). After stress-inducing acoustic trauma, reduced central compensation was also linked to reduced LTP and reduced levels of activity-dependent BDNF in hippocampal nerve endings and capillaries (Matt et al., 2018), seen here in untreated *low compensators* and *high compensators* treated with placebo (Figures 6, 7). A critical reduction of auditory input was suggested to hamper sufficient recruitment of activity-dependent BDNF to stimulate contrast amplification and distress levels (Matt et al., 2018; Knipper et al., 2020). Important in this context is that we, in line with previous studies analyzing *low compensators* vs. *high compensators* (Marchetta et al., 2020b), observed significantly lower activity-dependent BDNF levels in hippocampal regions in *low compensators* as compared to *high compensators*, which may mirror the low levels observed in *high compensators* after placebo-induced stress (Figure 7). This challenges the hypothesis that differences in auditory driving forces between *high* and *low compensators* may contribute to differences in hippocampal LTP through altered recruitment of BDNF-driven GR activation. Differences in activity-driven GR phosphorylation, in analogy to previous observations (Arango-Lievano et al., 2019), may be considered in future studies to be responsible for the observed different adaptive responses between *high* and *low compensators*.

Numerous studies support the notion that stress can impair central processing and task-induced response patterns through impaired neurovascular coupling (Sohal et al., 2009; Lee et al., 2015; Chen et al., 2017; Han et al., 2019). The BLEV reporter mouse, which allows for visualization and identification of activity-dependent *Bdnf* exon-IV and -VI derived expression, is an ideal tool to study molecular correlates of plasticity changes in the hippocampus (Matt et al., 2018). As *Bdnf* exon-IV-CFP is expressed in blood vessels and *Bdnf* exon-VI-YFP is expressed in synaptic nerve terminals, differences in *Bdnf* exon-IV and -VI expression observed between *high* and *low compensators* (Figures 7H,I) may reflect differences in hemodynamic hippocampal responses, as has previously been suggested (Marchetta et al., 2020b; Knipper et al., 2022). Considering that specific neuronal activity may tightly regulate blood flow (Hillman, 2014), it was hypothesized that a critically reduced driving force of auditory nerve fibers could lead to less recruitment of activity-driven *Bdnf* exon-VI-YFP transcripts in nerve endings and of *Bdnf* exon-IV-CFP expression in capillaries in *low compensators* in comparison to *high compensators* (Singer et al., 2018b; Marchetta et al., 2020b), which might reveal a chronically lower level of neurovascular coupling in *low compensators* and an acutely depressed level of neurovascular coupling due to stress in *high compensators*.

A loss of fast auditory processing, as seen in *high compensators* after treatment with placebo (Figure 5A), is here also linked to reduced levels of PV-positive interneuron (PV-IN) staining in the hippocampus (Figure 7J). Recently, stress has been shown to cause impaired neurovascular coupling and attenuation of cerebral blood flow through a reduction in PV-IN GABAergic signaling, possibly through altered nitric oxide-induced vasodilation (Czeh et al., 2015, 2018; Csabai et al., 2018; Han et al., 2019; Knipper et al., 2022). The differences between *high* and *low compensators* in *Bdnf* exon-IV-CFP expression in capillary vessels within the highly vascularized fissura region in conjunction with the altered PV-IN levels (Figure 7) may thus further point to differences in hemodynamic hippocampal responses.

The present study finally demonstrated that PDE9i treatment could ameliorate deficits in peripheral and central auditory processing in *high compensators* but not *low compensators*. Phosphodiesterase 9A (PDE9A) is a phosphodiesterase with high affinity for cGMP that is not only expressed in the brain (Lakics et al., 2010; Kelly, 2012) but also in the adult cochlea (Marchetta et al., 2020a). Preclinical studies have shown that a PDE9i can increase cGMP levels in the rat brain, enhance LTP, and improve memory function in rodent cognition tasks (van der Staay et al., 2008; Hutson et al., 2011; Kleiman et al., 2012; Kroker et al., 2012, 2014). This has also been shown for the PDE9i BAY 73-6691 used in the present study. BAY 73-6691 enhanced LTP in rat hippocampal slices (van der Staay et al., 2008) and stimulated protein synthesis-dependent late LTP in mouse hippocampal slices (Kroker et al., 2012). Furthermore, a PDE9i has been found to ameliorate auditory gating deficits in neurodegenerative diseases, such as Huntington's disease (Nagy et al., 2015). The present study provides new evidence that this PDE9i can ameliorate deficits in peripheral and central auditory processing, as well as in cognition under discrete conditions of preserved GC responsiveness in *high compensators*, but not when stress responses are blunted, as shown for *low compensators*. The results of previous studies suggesting that PDE9A inhibition can improve neurodegenerative diseases (e.g., Nagy et al., 2015; Sanders and Rajagopal, 2020; Sharma et al., 2020) may need to be reconsidered with the knowledge of this new finding.

**Figure 8:**
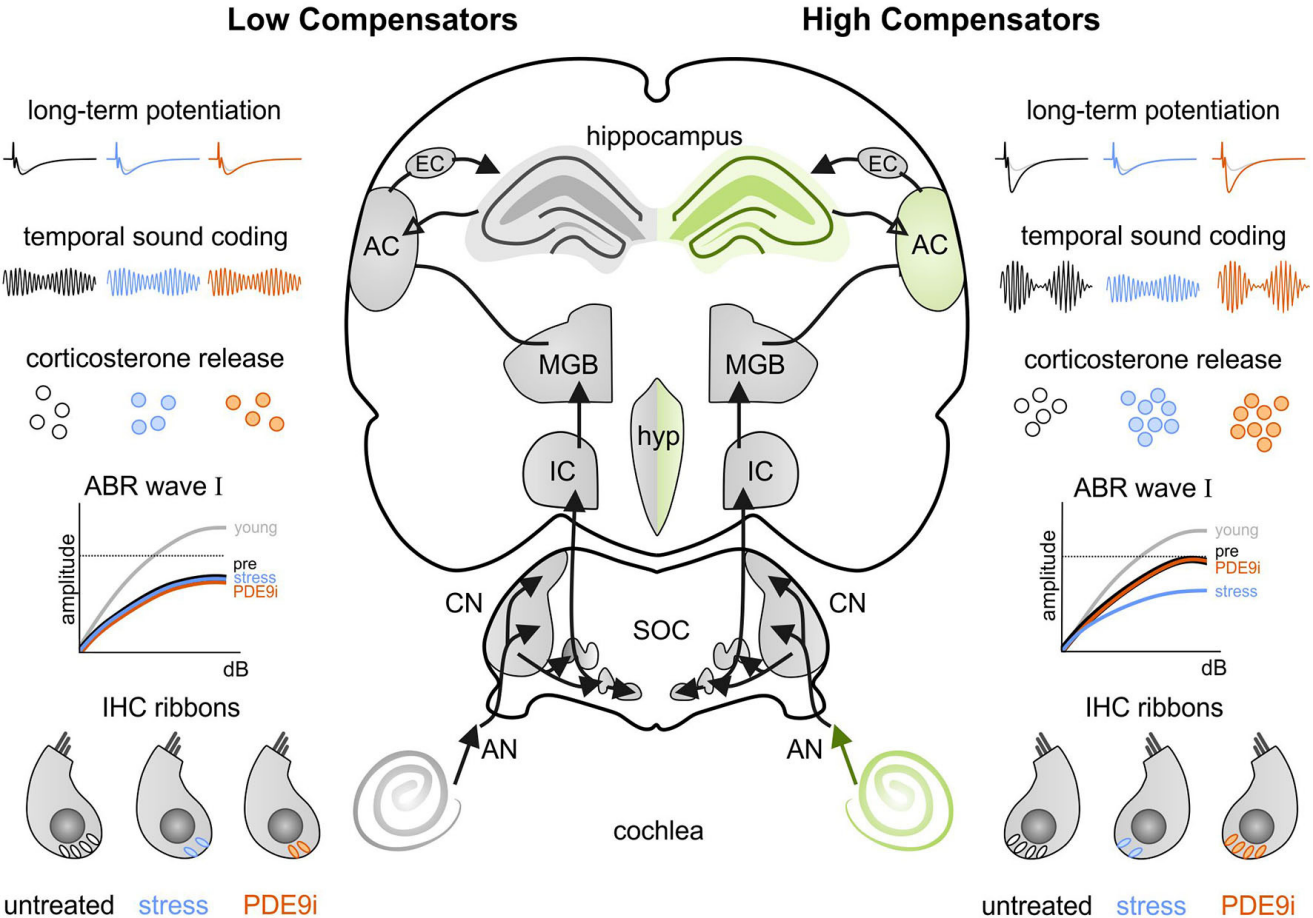
An abstract scheme of the mechanisms in the auditory pathway that are differentially affected by stress or PDE9i application in low compensators (the left side) and high compensators (the right side). Low compensators have a lower baseline corticosterone level (black, left) in comparison to high compensators (black, right). When stress due to repeated injections is applied (blue/orange), high compensators respond in a physiological way, with increased corticosterone release. Low compensators, on the other hand, do not have increased corticosterone levels after application of a stressor, showing characteristics of a blunted stress response. This blunted stress response prevents low compensators from reacting to both a stress-induced drop of long-term potentiation, temporal sound coding, auditory nerve response (ABR wave I), and inner hair cell ribbons, which we see in high compensators. Additionally, where treatment with a PDE9i is able to preserve all of these parameters in high compensators, the blunted stress response of low compensators prevents their restoration. ABR, auditory brainstem response; AC, auditory cortex; AN, auditory nerve; CN, cochlear nucleus; dB, decibels; EC, entorhinal cortex; hyp, hypothalamus; IC, inferior colliculus; IHC, inner hair cell; MGB, medial geniculate body; SOC, superior olivary complex.

In conclusion, central compensation following age-dependent cochlear synaptopathy can either be impaired upon a reduced stress response, as evident here in *low compensators* (Figure 8, left side), or it can be maintained in *high compensators*, who risk losing it under acute severe stress conditions (Figure 8, right side). In *low compensators*, stress induced by successive placebo injections cannot significantly reduce IHC ribbon numbers, ABR wave I amplitudes, ASSR, or LTP, or enhance PPF (Figure 8, left side). Under this condition of blunted stress response in *low compensators*, an elevation of cGMP levels by PDE9i cannot occur. In line with neural gain being stress and cGMP dependent in *high compensators*, features lost by stress can be restored through treatment with PDE9i.

Given the growing evidence of a link between hearing loss and dementia (Livingston et al., 2017; Griffiths et al., 2020; Montero-Odasso et al., 2020; Zhang et al., 2022), we sought to gain more insight into the nature of the relationship between cGMP signaling and stress receptors for central brain adaptation to environmental demands based on the present results. We suggest that balanced extra-hypothalamic stress control is a key signature that bridges auditory fiber processing with healthy cognition. We further emphasize that research into age-dependent cochlear synaptopathy and its future therapeutic intervention strategies by the pharmaceutical industry needs to consider individual states of stress response, particularly if aiming to use cognitive stimulators, e.g., PDE9i, as a therapeutic drug to overcome (auditory) cognitive decline.

## Data Availability Statement

The original contributions presented in the study are included in the article/Supplementary Material, further inquiries can be directed to the corresponding author/s.

## Ethics Statement

The animal study was reviewed and approved by University of Tübingen, Veterinary Care Unit, and the Animal Care and Ethics Committee of the regional board of the Federal State Government of Baden-Württemberg, Germany.

## Author Contributions

DS, MH, DC, PM, and CH performed the experiments. DS, MH, DC, CH, SF, LR, and WS performed data analyses. MK and PM performed microscopy. MK, LR, and WS contributed to conception and design of the study. PR critically revised the manuscript for intellectual content during this revision process and contributed to the interpretation of the data. All authors contributed to write, read, and approve the resubmitted version of the manuscript.

## Funding

This work was funded by the Deutsche Forschungsgemeinschaft (DFG, German Research Foundation) Research Training Group (Grant No. 335549539/GRK 2381), FOR 2060 project RU 713/3-2, SPP 1608 RU 316/12-1, KN 316/12-1, and the Sigmund Kiener Stiftung. Each source of funding provided a budget for personnel and material costs.

## Conflict of Interest

The authors declare that the research was conducted in the absence of any commercial or financial relationships that could be construed as a potential conflict of interest. The handling editor VS declared a past collaboration with the authors, LR, MK, and WS.

## Publisher's Note

All claims expressed in this article are solely those of the authors and do not necessarily represent those of their affiliated organizations, or those of the publisher, the editors and the reviewers. Any product that may be evaluated in this article, or claim that may be made by its manufacturer, is not guaranteed or endorsed by the publisher.

## Supplementary Material

Supplementary Figure 1 A schematic overview of ASSRs in mice. **(A)** The ASSRs were recorded with similar electrode positions as the ABR measurements, i.e., the active electrode placed below the pinna, the reference at the vertex and the grounding electrode close to the tail. **(B)** The presented amplitude-modulated stimulus contains a sine wave carrier frequency (CF) at 11.3 kHz, which is modulated by a modulation frequency (MF) of 512 Hz. The modulation of the CF is depicted by the dotted envelope function. **(C)** An example trace of a recorded signal in response to amplitude modulated stimuli; shown in the time-domain. **(D)** Conversion from the time- to the frequency-domain through Fast Fourier Transformation (FFT). Extraction of the signal at the noise floor, 512 Hz and the harmonics.

Supplementary Figure 2 Changes in hearing thresholds for click and noise ABR after placebo or PDE9i treatment. Neither **(A)** *low compensators* nor **(B)** *high compensators* showed changes in thresholds after treatment with placebo or PDE9i in response to click stimuli. The same result was observed in response to noise stimuli: thresholds of **(C)** *low compensators* and **(D)** *high compensators* remained unchanged with placebo and PDE9i. Mean ± SEM.

Supplementary Figure 3 ABR wave IV amplitude in *high* and *low* compensators before and after treatment with either placebo or PDE9i. **(A)** *High compensators* showed a significantly lower wave IV amplitude after placebo treatment, but **(B)** the wave IV amplitude of *high compensators* treated with PDE9i remained unchanged after treatment. **(C)** *Low compensators* showed no difference in wave IV amplitude after treatment with placebo or **(D)** with PDE9i. Mean ± SEM. ^*^*p* <0.05, repeated measures 2-way ANOVA.

Supplementary Figure 4 Input-output relationship between the fEPSP slope, fiber volley (FV) amplitude, and stimulus intensity. **(A)** Representative traces of fEPSPs with increasing stimulus intensities (from 25 μA to 150 μA in 25 μA steps). **(B)** In high-compensating animals, a significant increase in the fEPSP slope was observed after treatment with either placebo or PDE9i in comparison to their untreated controls at stimulus intensities between 50 and 100 μA. Low compensators treated with placebo and PDE9i also showed significantly increased fEPSP slopes in comparison to their untreated controls at stimulus intensities between 25 and 75 μA. In addition, PDE9i-treated *low compensators* had significantly increased fEPSP slopes at a stimulus intensity of 100 μA, and PDE9i-treated animals and placebo-treated animals were significantly different from one another at the stimulus intensity of 50 μA. **(C)** In *high compensators*, a significant increase in FV amplitude was observed after treatment with either placebo or PDE9i in comparison to untreated controls at stimulus intensities between 25 and 100 μA. In *low compensators*, the animals treated with PDE9i showed a significant increase in FV amplitude in comparison to untreated controls at stimulus intensities of 25 and 50 μA. *Low compensators* treated with placebo showed a significant increase in FV amplitude in comparison to untreated controls only at the stimulus intensity of 25 μA. No difference in FV amplitude between the treatment conditions of low compensators was observed. **(D)** Changes in fEPSP slopes were consistent with changes in FV amplitudes in all treatment groups of both *high* and *low compensators*. nt, no treatment; p, placebo. Mean ± SEM. ^***^*p* < 0.001, ^****^*p* < 0.0001, 2-way ANOVA.

Supplementary Figure 5 Paired-pulse facilitation (PPF) as an indicator of short-term plasticity. **(A)** Representative traces of fEPSPs with increasing interstimulus intervals (ISIs) at the stimulation strength equal to corresponding LTP recordings. Applied ISIs are indicated by the scale bar above: 10 ms, 20 ms, 50 ms, 100 ms, 200ms, 500 ms (not shown). **(B)** The analysis of PPF in *high compensators* showed a significantly lower paired-pulse ratio of the fEPSP2/fEPSP1 slope after PDE9i treatment in comparison to placebo treatment and to their untreated controls at ISIs of 10 and 20 ms, while placebo-treated high compensators did not differ significantly from their untreated controls. **(C)** *High compensators* treated with PDE9i had a significantly lower paired-pulse ratio of the fEPSP2/fEPSP1 amplitude in comparison to their untreated controls at ISIs of 10 and 20 ms, while placebo injection significantly increased the PPF amplitude ratio in *high compensators* at the same ISIs in comparison to their untreated controls. In addition, the paired-pulse ratio of the fEPSP2/fEPSP1 amplitude of placebo-treated and PDE9i-treated *high compensators* differed from each other at ISIs of 10, 20, and 50 ms. **(D)** The analysis of PPF in *low compensators* showed a significant effect of treatment on the paired-pulse ratio of the fEPSP2/fEPSP1 slope, but this was not significant in the *post hoc* test. **(E)** The paired-pulse ratio of the fEPSP2/fEPSP1 amplitude of *low compensators* treated with both placebo and PDE9i was significantly increased in comparison to the untreated controls at the ISI of 10 ms. No difference in the PPF amplitude ratio between the treatment conditions of *low compensators* was observed. nt, no treatment; p, placebo. Mean ± SEM. ^*^*p* < 0.05, ^**^*p* < 0.01, ^***^*p* < 0.001, ^****^*p* < 0.0001, 2-way ANOVA.

## Abbreviations

ABR: Auditory brainstem response
ACSF: artificial cerebrospinal fluid
ANOVA: analysis of variance
ASSR: auditory steady state response
BLEV: *Bdnf*-live-exon-visualization
BDNF/*Bdnf*: brain-derived neurotrophic factor
CFP/YFP: cyan- and yellow-fluorescent protein
cGMP: cyclic guanosine-monophosphate
FV: fiber volley
fEPSPs: field excitatory postsynaptic potentials
GC: glucocorticoid
GR: glucocorticoid receptor
HFS: high-frequency stimulation
IHC: inner hair cell
ISI: interstimulus interval
OHC: outer hair cell
IOR: input-output relationship
LTP: long-term potentiation
MD: mediodorsal thalamus
MR: mineralocorticoid receptor
PPF: paired-pulse facilitation
PV: parvalbumin
PV-IN: parvalbumin-positive interneuron
PDE9A: phosphodiesterase 9A
PDE9i: phosphodiesterase 9A inhibitor
PFC: prefrontal cortex
re thr: relative to threshold
SEM: standard error of the mean
SNR: signal-to-noise ratio.
