# Supplementary material for: Stress Affects Central Compensation of Neural Responses to Cochlear Synaptopathy in a cGMP-Dependent Way
Source: Front Neurosci. 2022 Jul 29;16:864706. doi: 10.3389/fnins.2022.864706 (PMC9372611; doi:10.3389/fnins.2022.864706)
Supplement: Supplementary file 1 [file Table_1.docx]

**Supplementary Table 1.** Statistical information of the results.

| Figure | Comparison | Statistical Test | Test Value | *p* - Value | Post-Hoc Test with *p* - Value | | *n* - Number |
| --- | --- | --- | --- | --- | --- | --- | --- |
| Fig. 1B | Click Thresholds | Linear regression | *R^2^* = 0.47 | *p* < 0.0001 |  |  | *n* = 58 |
| Fig. 1C | Noise Thresholds | Linear regression | *R^2^* = 0.33 | *p* < 0.0001 |  |  | *n* = 58 |
| Fig. 1D | ABR Wave I | Linear regression | *R^2^* = 0.22 | *p* = 0.0002 / *p* < 0.001 |  |  | *n* = 58 |
| Fig. 1E | ABR Wave IV | Linear regression | *R^2^* = 0.24 | *p* < 0.0001 |  |  | *n* = 58 |
| Fig. 2A | Click Thresholds | Mann-Whitney U test | U(30.05, 34.55) = 205.5 | *p* = 0.50 |  |  | low: *n* = 18  high: *n* = 26 |
| Fig. 2B | Noise Thresholds | Mann-Whitney U test | U(27, 27.35) = 200.5 | *p* = 0.70 |  |  | low: *n* = 18  high: *n* = 26 |
| Fig. 2C | 11 kHz Thresholds | Mann-Whitney U test | U(33, 43) = 197.5 | *p* = 0.39 |  |  | low: *n* = 18  high: *n* = 26 |
|  |  |  |  |  | **Bonferroni’s multiple comparisons test** | |  |
| Fig. 2D | fABR | 2-way ANOVA | F (1, 325) = 5.00 | *p* = 0.03 /  *p* < 0.05 | high pre vs low pre | *p* > 0.05 | low: *n* = 18  high: *n* = 26 |
|  |  |  |  |  | **Bonferroni’s multiple comparisons test** | |  |
| Fig. 2E | ASSR Response Growth | 2-way ANOVA | F (1, 546) = 13.41 | *p* = 0.0003 / *p* < 0.001 | high pre vs low pre | *p* < 0.05 for all SPL between 50 and 55 dB | low: *n* = 14  high: *n* = 20 |
| Fig. 3 | Corticosterone Levels | two-tailed Student’s t-test | *t(*35) = 2.42 | *p* = 0.02 /  *p* < 0.05 |  |  | low pre: *n* = 18  high pre: *n* = 19 |
|  |  |  |  |  | **Bonferroni’s multiple comparisons test** | |  |
|  |  | 1-way ANOVA | F (5, 63) = 6.79 | *p* < 0.001 | low pre vs low placebo | *p* > 0.05 | low pre: *n* = 18 |
|  |  |  |  |  | low pre vs low PDE9i | *p* > 0.05 | high pre: *n* = 19 |
|  |  |  |  |  | low placebo vs low PDE9i | *p* > 0.05 | low placebo: *n* = 6 |
|  |  |  |  |  | high pre vs high placebo | *p* < 0.01 | low PDE9i: *n* = 7 |
|  |  |  |  |  | high pre vs high PDE9i | *p* < 0.01 | high placebo: *n* = 10 |
|  |  |  |  |  | high placebo vs high PDE9i | *p* > 0.05 | high PDE9i: *n* = 9 |
|  |  |  |  |  | **Sidak’s multiple comparisons test** | |  |
| Fig. 4A | ABR Wave I Amplitude High Comp Placebo | Repeated measures 2-way ANOVA | F (1, 138) = 4.92 | *p* = 0.03 /  *p* < 0.05 | high pre vs post placebo | *p* > 0.05 | *n* = 20/10 |
| Fig. 4B | ABR Wave I Amplitude High Comp PDE9i | Repeated measures 2-way ANOVA | F (1, 124) = 0.15 | *p* = 0.70 |  |  | *n* = 22/11 |
| Fig. 4C | ABR Wave I Amplitude Low Comp Placebo | Repeated measures 2-way ANOVA | F (1, 44) = 2.04 | *p* = 0.16 |  |  | *n* = 8/4 |
| Fig. 4D | ABR Wave I Amplitude Low Comp PDE9i | Repeated measures 2-way ANOVA | F (1, 78) = 4.90 | *p* = 0.03 /  *p* < 0.05 |  |  | *n* = 12/6 |
| Fig. 4E | IHC Ribbons Apical Turn | two-tailed Student’s t-test | *t*(10) = 0.16 | *p* = 0.88 |  |  | low untreated: *n* = 6  high untreated: *n* = 6 |
|  |  | 1-way ANOVA | F (2, 18) = 0.01 | *p* = 0.99 |  |  | low untreated: *n* = 6  low placebo: *n* = 6  low PDE9i: *n* = 9 |
|  |  | 1-way ANOVA | F (2, 21) = 1.80 | *p* = 0.19 |  |  | high untreated: *n* = 6  high placebo: *n* = 10  high PDE9i: *n* = 8 |
| Fig. 4F | IHC Ribbons Medial Turn | two-tailed Student’s t-test | *t*(10) = 0.73 | *p* = 0.48 |  |  | low untreated: *n* = 6  high untreated: *n* = 6 |
|  |  |  |  |  | **Sidak’s multiple comparisons test** | |  |
|  |  | 1-way ANOVA | F (2, 18) = 1.19 | *p* = 0.33 |  |  | low untreated: *n* = 6  low placebo: *n* = 6  low PDE9i: *n* = 9 |
|  |  | 1-way ANOVA | F (2, 21) = 3.54 | *p* = 0.048 / *p* < 0.05 | high untreated vs high placebo  high untreated vs high PDE9i  high placebo vs high PDE9i | *p* > 0.05  *p* > 0.05  *p* < 0.05 | high untreated: *n* = 6  high placebo: *n* = 10  high PDE9i: *n* = 8 |
| Fig. 4G | IHC Ribbons Midbasal Turn | two-tailed Student’s t-test | *t*(10) = 0.89 | *p* = 0.40 |  |  | low untreated: *n* = 6  high untreated: *n* = 6 |
|  |  |  |  |  | **Sidak’s multiple comparisons test** | |  |
|  |  | 1-way ANOVA | F (2, 18) = 3.15 | *p* = 0.07 |  |  | low untreated: *n* = 6  low placebo: *n* = 6  low PDE9i: *n* = 9 |
|  |  | 1-way ANOVA | F (2, 21) = 5.78 | *p* = 0.01 /  *p* < 0.05 | high untreated vs high placebo  high untreated vs high PDE9i  high placebo vs high PDE9i | *p* > 0.05  *p* > 0.05  *p* < 0.05 | high untreated: *n* = 6  high placebo: *n* = 10  high PDE9i: *n* = 8 |
|  |  |  |  |  | **Sidak’s multiple comparisons test** | |  |
| Fig. 5A | ASSR High Comp Placebo | Repeated measures 2-way ANOVA | F (1, 122) = 44.30 | *p* < 0.0001 | high pre vs post placebo | *p* < 0.05 for all SPL between 35 and 60 dB | *n* = 10 |
| Fig. 5B | ASSR High Comp PDE9i | Repeated measures 2-way ANOVA | F (1, 106) = 1.52 | *p* = 0.22 |  |  | *n* = 10 |
| Fig. 5C | ASSR Low Comp Placebo | Repeated measures 2-way ANOVA | F (1, 56) = 1.05 | *p* = 0.31 |  |  | *n* = 6 |
| Fig. 5D | ASSR Low Comp PDE9i | Repeated measures 2-way ANOVA | F (1, 86) = 0.01 | *p* = 0.94 |  |  | *n* = 8 |
| Fig. 6B | LTP | 1-way ANOVA | F (6, 49) = 11.17, | *p* < 0.0001 |  |  |  |
| Fig. 6C | LTP | two-tailed Student's t-test | *t*(12) = 3.17 | *p* = 0.008 / *p* < 0.01 |  |  | low untreated: *n* = 2/5  high untreated: *n* = 4/9 |
|  |  |  |  |  | **two-stage linear step-up procedure of Benjamini, Krieger, and Yekutieli** | |  |
|  |  | 1-way ANOVA | F (2, 18) = 0.81 | *p* = 0.46 |  |  | low untreated: *n* = 2/5  low placebo: *n* = 2/7  low PDE9i: *n* = 3/9 |
|  |  | 1-way ANOVA | F (2, 23) = 8.24 | *p* = 0.002 / *p* < 0.01 | high untreated vs high placebo  high untreated vs high PDE9i  high placebo vs high PDE9i | *p* < 0.001  *p* > 0.05  *p* < 0.05 | high untreated: *n* = 4/9  high placebo: *n* = 3/9  high PDE9i: *n* = 2/8 |
| Fig. 6D | PPF Slope at 20 ms | Mann-Whitney U test | U(1.478, 1.002) = 11 | *p* = 0.15 |  |  | low untreated: *n* = 2/5  high untreated: *n* = 4/9 |
|  |  | Kruskal-Wallis test | H (2) = 1.12 | *p* = 0.59 |  |  | low untreated: *n* = 2/5  low placebo: *n* = 2/7  low PDE9i*: n* = 3/9 |
|  |  | Kruskal-Wallis test | H (2) = 2.78 | *p* = 0.25 |  |  | high untreated: *n* = 4/9  high placebo: *n* = 3/9  high PDE9i: *n* = 2/8 |
| Fig. 6E | PPF Amplitude at 20 ms | two-tailed Student's t-test | *t(*12) = 2.58 | *p* = 0.02 /  *p* < 0.05 |  |  | low untreated: *n* = 2/5  high untreated: *n* = 4/9 |
|  |  |  |  |  | **Sidak’s multiple comparisons test** | |  |
|  |  | 1-way ANOVA | F (2, 18) = 1.10 | *p* = 0.36 |  |  | low untreated: *n* = 2/5  low placebo: *n* = 2/7  low PDE9i: *n* = 3/9 |
|  |  | 1-way ANOVA | F (2, 24) = 5.19 | *p* = 0.01 /  *p* < 0.05 | high untreated vs high placebo  high untreated vs high PDE9i  high placebo vs high PDE9i | *p* > 0.05  *p* > 0.05  *p* < 0.05 | high untreated: *n* = 4/9  high placebo: *n* = 3/9  high PDE9i: *n* = 2/8 |
| Fig. 7H | *Bdnf* exon-IV-CFP | Mann-Whitney U test | U(3.255, 4.197) = 5 | *p* = 0.07 |  |  | low untreated: *n* = 5/8  high untreated: *n* = 2/4 |
|  |  |  |  |  | **Dunn's multiple comparisons test** | |  |
|  |  | Kruskal-Wallis test | H (2) = 1.71 | *p* = 0.43 |  |  | low untreated: *n* = 5/8  low placebo: *n* = 5/8  low PDE9i: *n* = 4/7 |
|  |  | Kruskal-Wallis test | H (2) = 9.42 | *p* = 0.009 / *p* < 0.01 | high untreated vs high placebo  high untreated vs high PDE9i  high placebo vs high PDE9i | *p* > 0.05  *p* > 0.05  *p* < 0.01 | high untreated: *n* = 2/4  high placebo: *n* = 2/4  high PDE9i: *n* = 4/8 |
| Fig. 7I | *Bdnf* exon-VI-YFP | Mann-Whitney U test | U(2.749, 5.218) = 0 | *p* = 0.004 / *p* < 0.01 |  |  | low untreated: *n* = 5/8  high untreated: *n* = 2/4 |
|  |  |  |  |  | **Dunn's multiple comparisons test** | |  |
|  |  | Kruskal-Wallis test | H (2) = 4.83 | *p* = 0.09 |  |  | low untreated: *n* = 5/8  low placebo: *n* = 5/8  low PDE9i: *n* = 4/8 |
|  |  | Kruskal-Wallis test | H (2) = 6.05 | *p* = 0.049 / *p* < 0.05 | high untreated vs high placebo  high untreated vs high PDE9i  high placebo vs high PDE9i | *p* > 0.05  *p* > 0.05  *(*) p= 0.07; p* > 0.05 | high untreated: *n* = 2/4  high placebo: *n* = 2/4  high PDE9i: *n* = 4/8 |
| Fig. 7J | Parvalbumin | Mann-Whitney U test | U(5.550, 7.208) = 13 | *p* = 0.68 |  |  | low untreated: *n* = 5/8  high untreated: *n* = 2/4 |
|  |  |  |  |  | **Dunn's multiple comparisons test** | |  |
|  |  | Kruskal-Wallis test | H (2) = 0.25 | *p* = 0.88 |  |  | low untreated: *n* = 5/8  low placebo: *n* = 4/8  low PDE9i: *n* = 4/8 |
|  |  | Kruskal-Wallis test | H (2) = 8.82 | *p* = 0.01 /  *p* < 0.05 | high untreated vs high placebo  high untreated vs high PDE9i  high placebo vs high PDE9i | *p* > 0.05  *p* > 0.05  *p* < 0.01 | high untreated: *n* = 2/4  high placebo: *n* = 2/4  high PDE9i: *n* = 4/8 |
| Fig. S2A | Click Thresholds Low Comp | 1-way ANOVA | F (3, 24) = 0.19 | *p* = 0.91 |  |  | placebo: *n* = 6  PDE9i: *n* = 8 |
| Fig. S2B | Click Thresholds High Comp | 1-way ANOVA | F (3, 36) = 0.63 | *p* = 0.60 |  |  | placebo: *n* = 9  PDE9i: *n* = 11 |
| Fig. S2C | Noise Thresholds Low Comp | Kruskal-Wallis test | H (3) = 0.49 | *p* = 0.92 |  |  | placebo: *n* = 6  PDE9i: *n* = 8 |
| Fig. S2D | Noise Thresholds High Comp | Kruskal-Wallis test | H (3) = 3.47 | *p* = 0.32 |  |  | placebo: *n* = 9  PDE9i: *n* = 11 |
| Fig. S3A | ABR Wave IV Amplitude High Comp Placebo | Repeated measures 2-way ANOVA | F (1, 123) = 5.72 | *p* = 0.02 /  *p* < 0.05 |  |  | *n* = 20/10 |
| Fig. S3B | ABR Wave IV Amplitude High Comp PDE9i | Repeated measures 2-way ANOVA | F (1, 127) = 2.49 | *p* = 0.12 |  |  | *n* = 22/11 |
| Fig. S3C | ABR Wave IV Amplitude Low Comp Placebo | Repeated measures 2-way ANOVA | F (1, 47) = 0.06 | *p* = 0.82 |  |  | *n* = 8/4 |
| Fig. S3D | ABR Wave IV Amplitude Low Comp PDE9i | Repeated measures 2-way ANOVA | F (1, 77) = 2.27 | *p* = 0.14 |  |  | *n* = 12/6 |
|  |  |  |  |  | **two-stage linear step-up procedure of Benjamini, Krieger, and Yekutieli** | |  |
| Fig. S4B | IOR fEPSP Slope High Comp | 2-way ANOVA | F (2, 144) = 10.97 | *p* < 0.0001 | high untreated vs high placebo | *p* < 0.05 for intensities between 50 and 100 µA | high untreated: *n* = 4/9 |
|  |  |  |  |  | high untreated vs high PDE9i | *p* < 0.05 for intensities between 50 and 100 µA | high placebo: *n* = 3/9 |
|  |  |  |  |  | high placebo vs high PDE9i | *p* > 0.05 | high PDE9i: *n* = 2/8 |
|  | IOR fEPSP Slope Low Comp | 2-way ANOVA | F (2, 108) = 17.72 | *p* < 0.0001 | low untreated vs low placebo | *p* < 0.05 for intensities between 25 and 75 µA | low untreated: *n* = 2/5 |
|  |  |  |  |  | low untreated vs low PDE9i | *p* < 0.05 for intensities between 25 and 125 µA | low placebo: *n* = 2/7 |
|  |  |  |  |  | low placebo vs low PDE9i | *p* < 0.05 for 50 µA | low PDE9i: *n* = 3/9 |
| Fig. S4C | IOR Fiber Volley Amplitude High Comp | 2-way ANOVA | F (2, 144) = 59.17 | *p* < 0.0001 | high untreated vs high placebo | *p* < 0.05 for intensities between 25 and 125 µA | high untreated: *n* = 4/9 |
|  |  |  |  |  | high untreated vs high PDE9i | *p* < 0.05 for intensities between 25 and 125 µA | high placebo: *n* = 3/9 |
|  |  |  |  |  | high placebo vs high PDE9i | *p* > 0.05 | high PDE9i: *n* = 2/8 |
|  | IOR Fiber Volley Amplitude Low Comp | 2-way ANOVA | F (2, 108) = 7.71 | *p* = 0.0007 / *p* < 0.001 | low untreated vs low placebo | *p* < 0.05 for intensities between 25 and 50 µA | low untreated: *n* = 2/5 |
|  |  |  |  |  | low untreated vs low PDE9i | *p* < 0.05 for 25 µA | low placebo: *n* = 2/7 |
|  |  |  |  |  | low placebo vs low PDE9i | *p* > 0.05 | low PDE9i: *n* = 3/9 |
| Fig. S4D | fEPSP Slope vs. Fiber Volley Amplitude High Comp | Difference between regression lines (slopes) | F (2, 155) = 0.45 | *p* = 0.64 |  |  | high untreated: *n* = 4/9  high placebo: *n* = 3/9  high PDE9i: *n* = 2/8 |
|  | fEPSP Slope vs. Fiber Volley Amplitude Low Comp | Difference between regression lines (slopes) | F (2, 120) = 0.04 | *p* = 0.96 |  |  | low untreated: *n* = 2/5  low placebo: *n* = 2/7  low PDE9i: *n* = 3/9 |
|  |  |  |  |  | **two-stage linear step-up procedure of Benjamini, Krieger, and Yekutieli** | |  |
| Fig. S5B | PPF Slope High Comp | 2-way ANOVA | F (2, 144) = 9.07 | *p* = 0.0002 / *p* < 0.001 | high untreated vs high placebo | *p* > 0.05 | high untreated: *n* = 4/9 |
|  |  |  |  |  | high untreated vs high PDE9i | *p* < 0.05 for all ISIs between 10 and 20 ms | high placebo: *n* = 3/9 |
|  |  |  |  |  | high placebo vs high PDE9i | *p* < 0.05 for all ISIs between 10 and 20 ms | high PDE9i: *n* = 2/8 |
| Fig. S5C | PPF Amplitude High Comp | 2-way ANOVA | F (2, 144) =24.04 | *p* < 0.0001 | high untreated vs high placebo | *p* < 0.05 for all ISIs between 10 and 20 ms | high untreated: *n* = 4/9 |
|  |  |  |  |  | high untreated vs high PDE9i | *p* < 0.05 for all ISIs between 10 and 20 ms | high placebo: *n* = 3/9 |
|  |  |  |  |  | high placebo vs high PDE9i | *p* < 0.05 for all ISIs between 10 and 50 ms | high PDE9i: *n* = 2/8 |
| Fig. S5D | PPF Slope Low Comp | 2-way ANOVA | F (2, 108) = 4.40 | *p* = 0.02 /  *p* < 0.05 | low untreated vs low placebo  low untreated vs low PDE9i  low placebo vs low PDE9i | *p* > 0.05  *p* > 0.05  *p* > 0.05 | low untreated: *n* = 2/5  low placebo: *n* = 2/7  low PDE9i: *n* = 3/9 |
| Fig. S5E | PPF Amplitude Low Comp | 2-way ANOVA | F (2, 108) = 6.09 | *p* = 0.003 / *p* < 0.01 | low untreated vs low placebo | *p* < 0.05 for 10 ms | low untreated: *n* = 2/5  low placebo: *n* = 2/7  low PDE9i: *n* = 3/9 |
|  |  |  |  |  | low untreated vs low PDE9i | *p* < 0.05 for 10 ms |  |
|  |  |  |  |  | low placebo vs low PDE9i | *p* > 0.05 |  |
