# Supplementary figures and images for: Stress Affects Central Compensation of Neural Responses to Cochlear Synaptopathy in a cGMP-Dependent Way
Source: Front Neurosci. 2022 Jul 29;16:864706. doi: 10.3389/fnins.2022.864706 (PMC9372611; doi:10.3389/fnins.2022.864706)

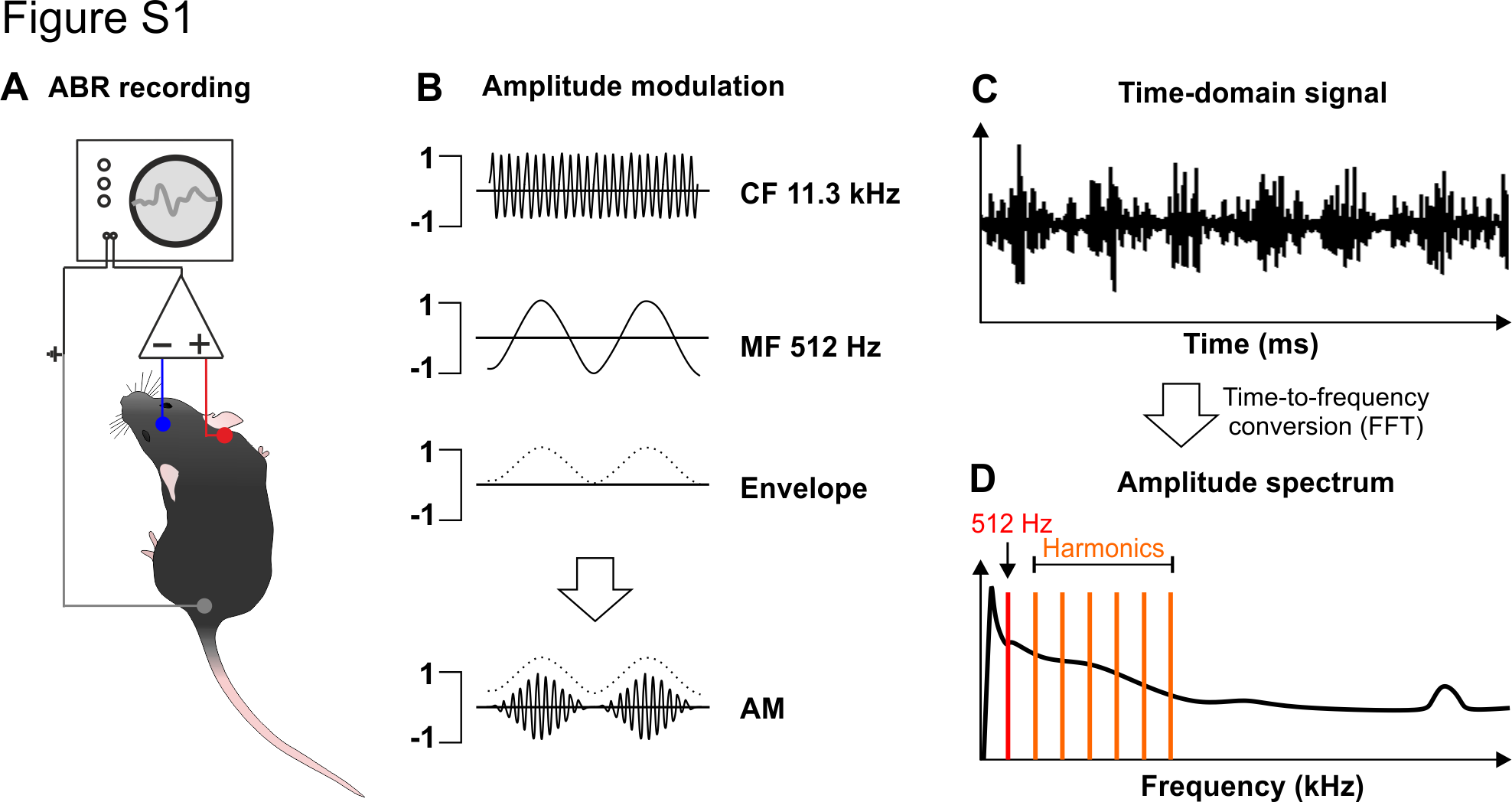

Supplement: Supplementary Figure 1 — A schematic overview of ASSRs in mice. (A) The ASSRs were recorded with similar electrode positions as the ABR measurements, i.e., the active electrode placed below the pinna, the reference at the vertex and the grounding electrode close to the tail. (B) The presented amplitude-modulated stimulus contains a sine wave carrier frequency (CF) at 11.3 kHz, which is modulated by a modulation frequency (MF) of 512 Hz. The modulation of the CF is depicted by the dotted envelope function. (C) An example trace of a recorded signal in response to amplitude modulated stimuli; shown in the time-domain. (D) Conversion from the time- to the frequency-domain through Fast Fourier Transformation (FFT). Extraction of the signal at the noise floor, 512 Hz and the harmonics. [file Image_1.TIF]

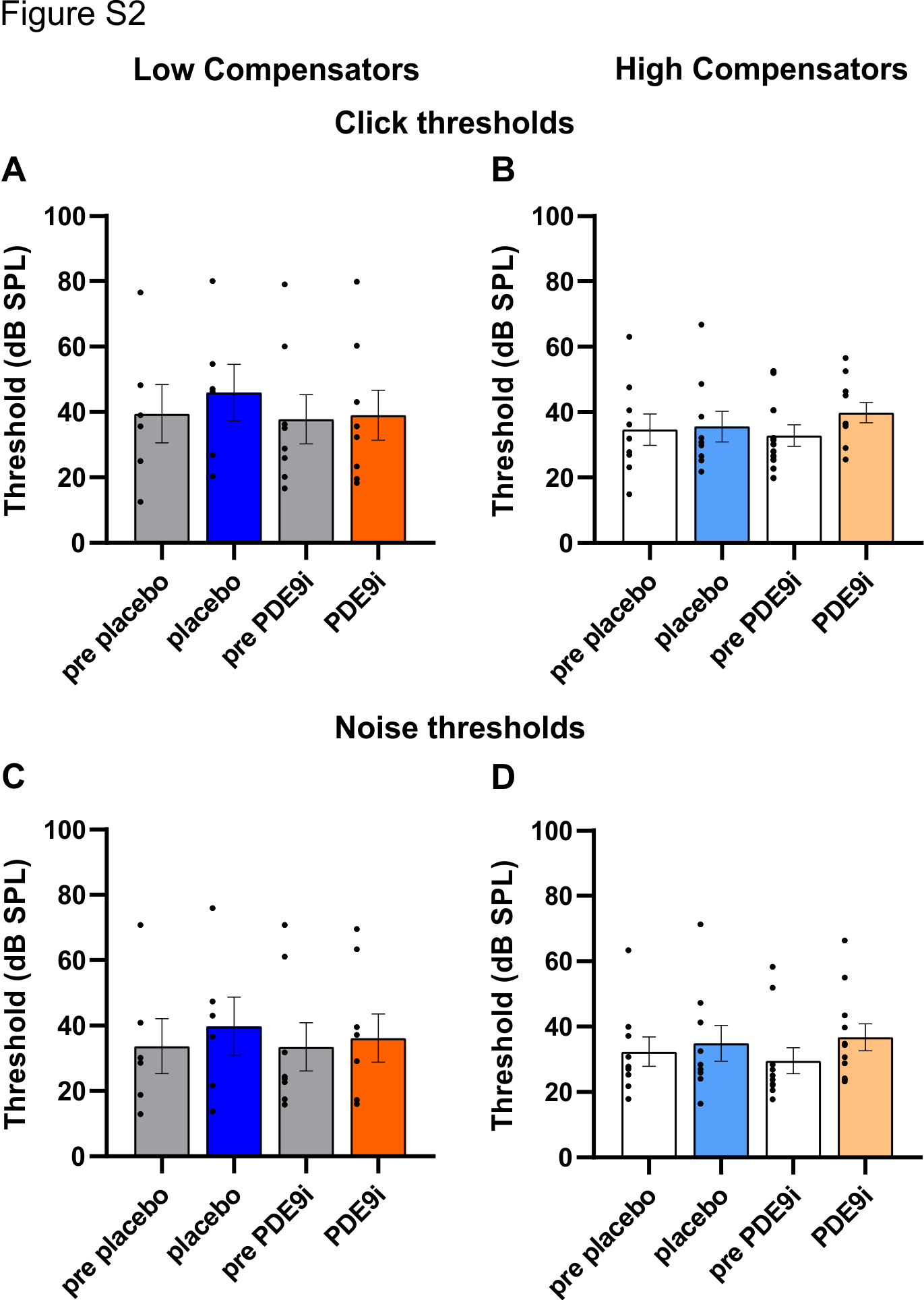

Supplement: Supplementary Figure 2 — Changes in hearing thresholds for click and noise ABR after placebo or PDE9i treatment. Neither (A) low compensators nor (B) high compensators showed changes in thresholds after treatment with placebo or PDE9i in response to click stimuli. The same result was observed in response to noise stimuli: thresholds of (C) low compensators and (D) high compensators remained unchanged with placebo and PDE9i. Mean ± SEM. [file Image_2.TIF]

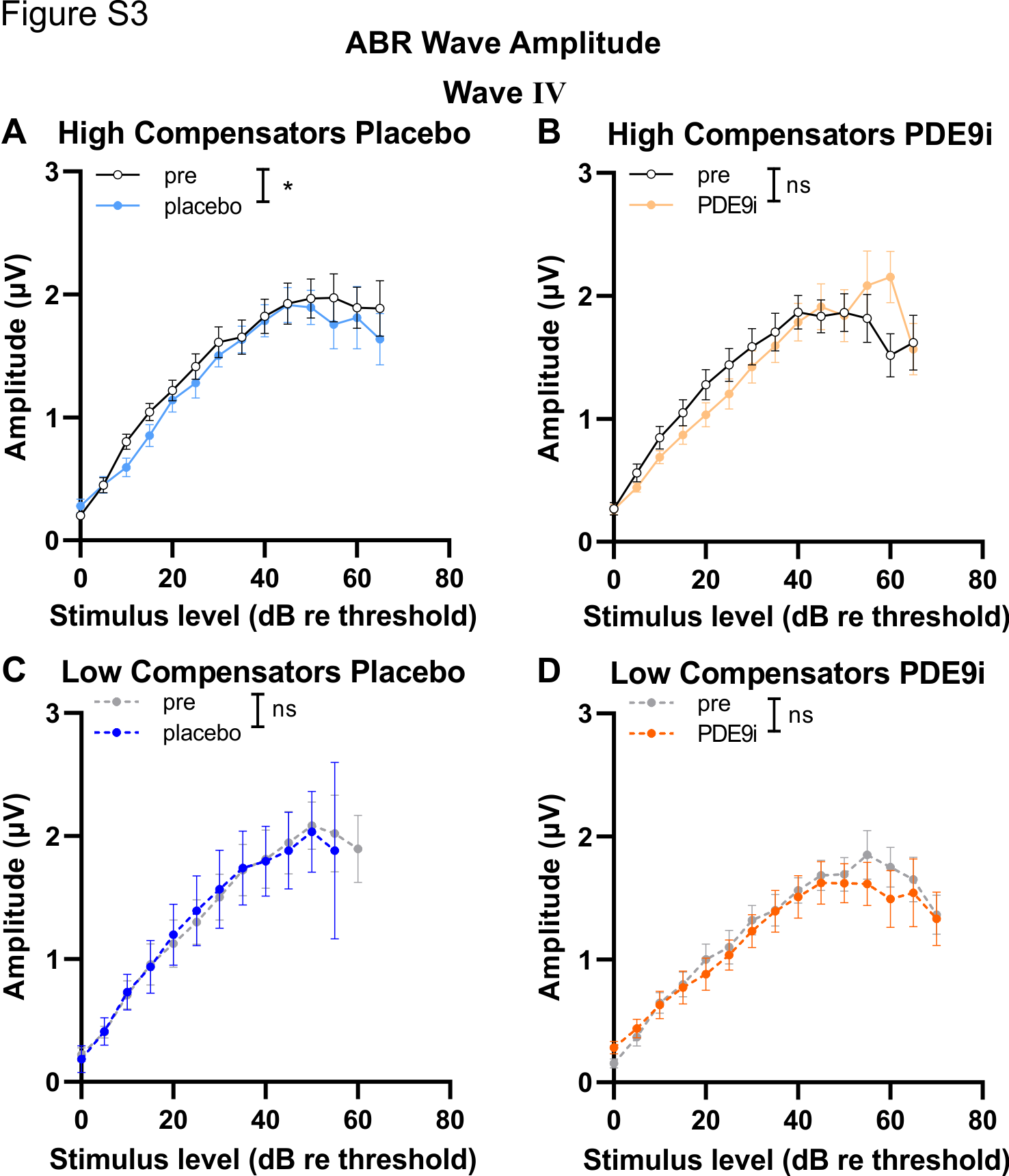

Supplement: Supplementary Figure 3 — ABR wave IV amplitude in high and low compensators before and after treatment with either placebo or PDE9i. (A) High compensators showed a significantly lower wave IV amplitude after placebo treatment, but (B) the wave IV amplitude of high compensators treated with PDE9i remained unchanged after treatment. (C) Low compensators showed no difference in wave IV amplitude after treatment with placebo or (D) with PDE9i. Mean ± SEM. *p <0.05, repeated measures 2-way ANOVA. [file Image_3.TIF]

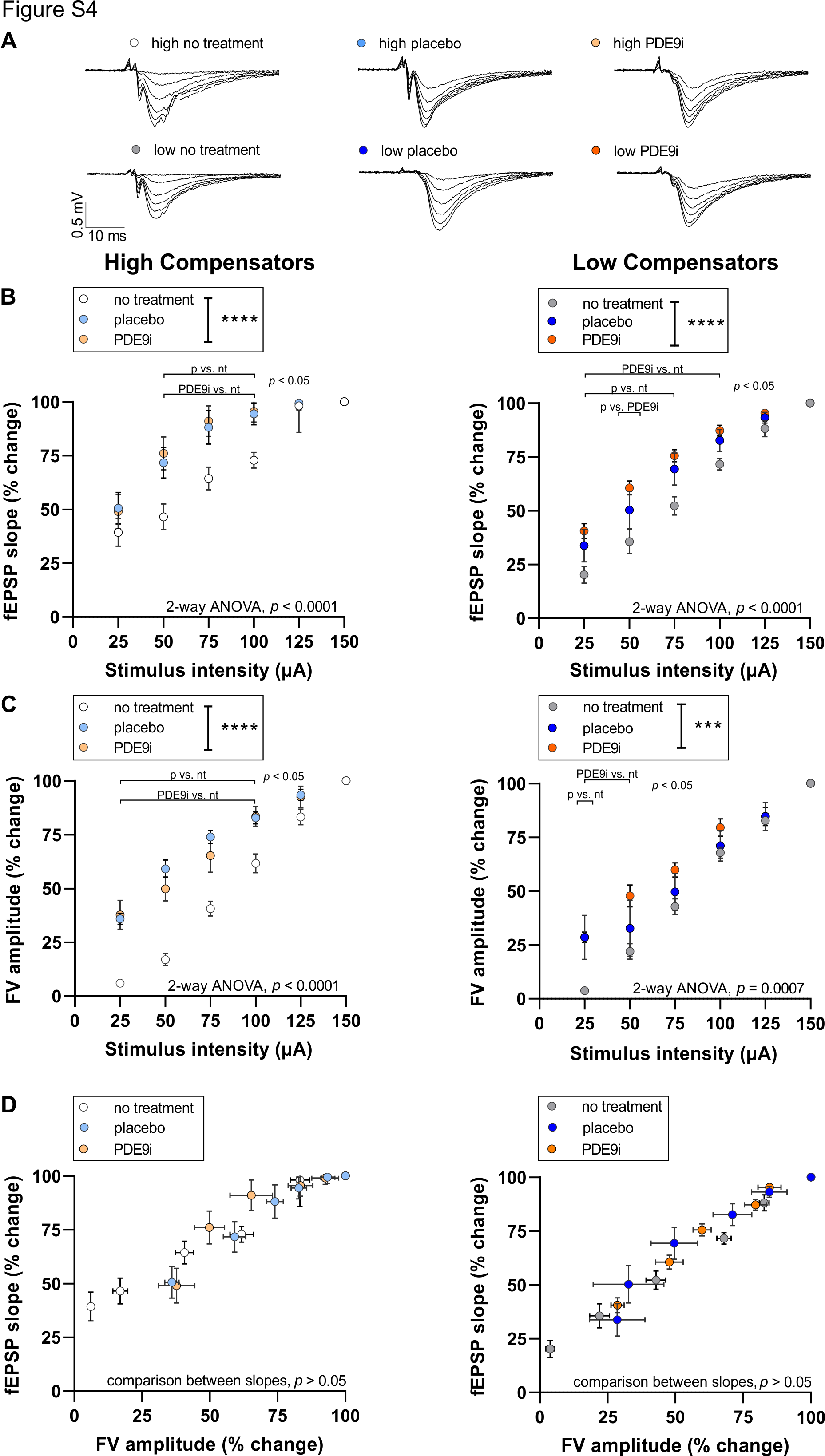

Supplement: Supplementary Figure 4 — Input-output relationship between the fEPSP slope, fiber volley (FV) amplitude, and stimulus intensity. (A) Representative traces of fEPSPs with increasing stimulus intensities (from 25 μA to 150 μA in 25 μA steps). (B) In high-compensating animals, a significant increase in the fEPSP slope was observed after treatment with either placebo or PDE9i in comparison to their untreated controls at stimulus intensities between 50 and 100 μA. Low compensators treated with placebo and PDE9i also showed significantly increased fEPSP slopes in comparison to their untreated controls at stimulus intensities between 25 and 75 μA. In addition, PDE9i-treated low compensators had significantly increased fEPSP slopes at a stimulus intensity of 100 μA, and PDE9i-treated animals and placebo-treated animals were significantly different from one another at the stimulus intensity of 50 μA. (C) In high compensators, a significant increase in FV amplitude was observed after treatment with either placebo or PDE9i in comparison to untreated controls at stimulus intensities between 25 and 100 μA. In low compensators, the animals treated with PDE9i showed a significant increase in FV amplitude in comparison to untreated controls at stimulus intensities of 25 and 50 μA. Low compensators treated with placebo showed a significant increase in FV amplitude in comparison to untreated controls only at the stimulus intensity of 25 μA. No difference in FV amplitude between the treatment conditions of low compensators was observed. (D) Changes in fEPSP slopes were consistent with changes in FV amplitudes in all treatment groups of both high and low compensators. nt, no treatment; p, placebo. Mean ± SEM. ***p < 0.001, ****p < 0.0001, 2-way ANOVA. [file Image_4.TIF]

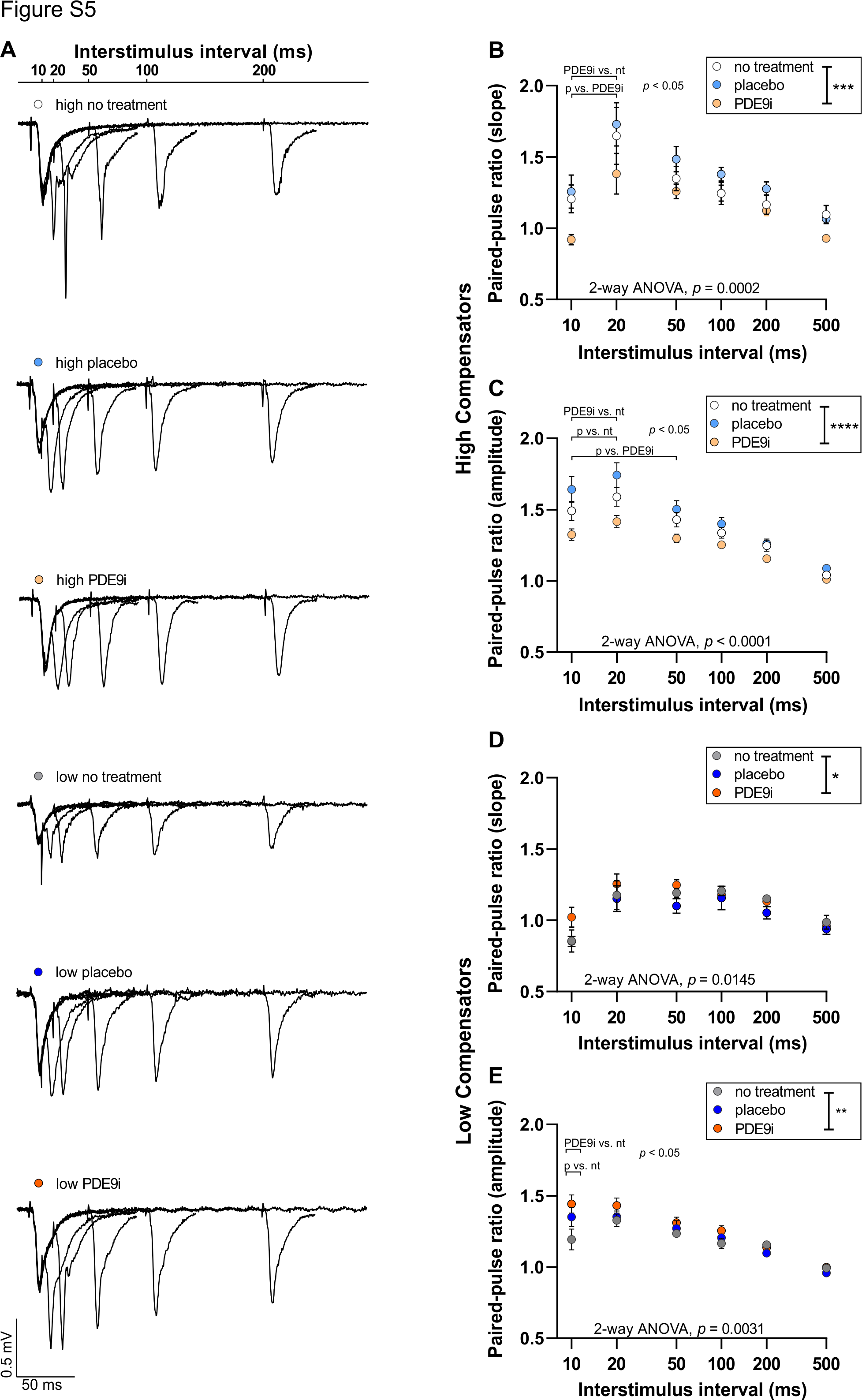

Supplement: Supplementary Figure 5 — Paired-pulse facilitation (PPF) as an indicator of short-term plasticity. (A) Representative traces of fEPSPs with increasing interstimulus intervals (ISIs) at the stimulation strength equal to corresponding LTP recordings. Applied ISIs are indicated by the scale bar above: 10 ms, 20 ms, 50 ms, 100 ms, 200ms, 500 ms (not shown). (B) The analysis of PPF in high compensators showed a significantly lower paired-pulse ratio of the fEPSP2/fEPSP1 slope after PDE9i treatment in comparison to placebo treatment and to their untreated controls at ISIs of 10 and 20 ms, while placebo-treated high compensators did not differ significantly from their untreated controls. (C) High compensators treated with PDE9i had a significantly lower paired-pulse ratio of the fEPSP2/fEPSP1 amplitude in comparison to their untreated controls at ISIs of 10 and 20 ms, while placebo injection significantly increased the PPF amplitude ratio in high compensators at the same ISIs in comparison to their untreated controls. In addition, the paired-pulse ratio of the fEPSP2/fEPSP1 amplitude of placebo-treated and PDE9i-treated high compensators differed from each other at ISIs of 10, 20, and 50 ms. (D) The analysis of PPF in low compensators showed a significant effect of treatment on the paired-pulse ratio of the fEPSP2/fEPSP1 slope, but this was not significant in the post hoc test. (E) The paired-pulse ratio of the fEPSP2/fEPSP1 amplitude of low compensators treated with both placebo and PDE9i was significantly increased in comparison to the untreated controls at the ISI of 10 ms. No difference in the PPF amplitude ratio between the treatment conditions of low compensators was observed. nt, no treatment; p, placebo. Mean ± SEM. *p < 0.05, **p < 0.01, ***p < 0.001, ****p < 0.0001, 2-way ANOVA. [file Image_5.TIF]
